# STAT3 activation in large granular lymphocyte leukemia is associated with cytokine signaling and DNA hypermethylation

**DOI:** 10.1038/s41375-021-01296-0

**Published:** 2021-06-01

**Authors:** Daehong Kim, Giljun Park, Jani Huuhtanen, Bishwa Ghimire, Hanna Rajala, Richard Moriggl, Wing C. Chan, Matti Kankainen, Mikko Myllymäki, Satu Mustjoki

**Affiliations:** 1 Hematology Research Unit Helsinki, University of Helsinki and Department of Hematology, Helsinki University Hospital Comprehensive Cancer Center, Helsinki, Finland; 2 Translational Immunology Research Program and Department of Clinical Chemistry and Hematology, University of Helsinki, Helsinki, Finland; 3 Institute for Molecular Medicine Finland, University of Helsinki, Helsinki, Finland; 4 Institute of Animal Breeding and Genetics, University of Veterinary Medicine Vienna, Vienna, Austria; 5 Department of Pathology, City of Hope National Medical Center, Duarte, CA USA; 6 Department of Medical and Clinical Genetics, University of Helsinki and Helsinki University Hospital, Helsinki, Finland; 7 iCAN Digital Precision Cancer Medicine Flagship, Helsinki, Finland

**Keywords:** Leukaemia, Lymphocytes

## Abstract

Large granular lymphocyte leukemia (LGLL) is characterized by somatic gain-of-function *STAT3* mutations. However, the functional effects of *STAT3* mutations on primary LGLL cells have not been studied in detail. In this study, we show that CD8+ T cells isolated from *STAT3* mutated LGLL patients have high protein levels of epigenetic regulators, such as DNMT1, and are characterized by global hypermethylation. Correspondingly, treatment of healthy CD8+ T cells with IL-6, IL-15, and/or MCP-1 cytokines resulted in STAT3 activation, increased DNMT1, EZH2, c-MYC, l-MYC, MAX, and NFκB levels, increased DNA methylation, and increased oxidative stress. Similar results were discovered in KAI3 NK cells overexpressing gain-of-function *STAT3*^Y640F^ and *STAT3*^G618R^ mutants compared to KAI3 NK cells overexpressing *STAT3*^WT^. Our results also confirm that STAT3 forms a direct complex with DNMT1, EZH2, and HDAC1. In *STAT3* mutated LGLL cells, DNA methyltransferase (DNMT) inhibitor azacitidine abrogated the activation of STAT3 via restored SHP1 expression. In conclusion, *STAT3* mutations cause DNA hypermethylation resulting in sensitivity to DNMT inhibitors, which could be considered as a novel treatment option for LGLL patients with resistance to standard treatments.

## Introduction

Large granular lymphocyte leukemia (LGLL) is a rare chronic lymphoproliferative disorder, which in over 80% of the cases results from abnormal clonal expansion of CD3 + CD8 + cytotoxic T cells [1]. Less frequently, LGLL originates from the expansion of natural killer (NK) cells (chronic lymphoproliferative disorder of NK cells, CLPD-NK). Both T-cell LGLL and CLPD-NK are chronic and indolent diseases, but instances of aggressive leukemias have also been reported [2].

Somatic gain-of-function (GOF) *STAT3* mutations are a hallmark of LGLL and occur in both T and NK forms of the disease [3–8]. LGLL patients with *STAT3* mutations have more often neutropenia and rheumatoid arthritis (RA) [3, 9, 10], and mutations may also confer reduced overall survival [5]. Although STAT3 activation, irrespective of mutation status, is one of the key features and a known mediator of cytokine signaling and inflammation in LGLL [1, 11–13], the functional consequences of STAT3 activation in LGLL remain to be fully elucidated.

Cytokines induce differentiation and proliferation of lymphocytes, and aberrant cytokine signaling can be observed in many inflammatory conditions [14]. Interleukin-6 (IL-6) is elevated in RA leading to JAK/STAT3 signaling activation, and IL-6 and IL-6 receptor inhibitors are used in RA treatment [15]. In addition to the well-established role in chronic inflammatory diseases, cytokine dysregulation may also drive tumor initiation and progression [16]. In LGLL mouse model, excessive IL-15 induced DNMT3b upregulation and global DNA hypermethylation, and IL-15 may drive LGLL formation [17–19].

As the role of STAT3 in regulating DNA methylation in LGLL is currently unknown, we investigated whether inflammatory cytokine stimulation and STAT3 activation modify the epigenetic machinery in CD8+ T cells and could thereby affect disease pathogenesis in LGLL. Using LGLL patients samples, healthy human CD8+ T cells, and KAI3 NK cell line, we show that cytokine stimulation or overexpression of GOF *STAT3* variants lead to increased protein levels of epigenetic regulators, increased global DNA methylation levels, and increased reactive oxygen species (ROS) production. Treatment with DNMT inhibitor abrogated STAT3 mediated changes in SHP1 dependent manner, paving the way for novel targeted therapeutic approaches.

## Materials and methods

### Materials and methods

More detailed descriptions of the methods and analyses are available in the Supplementary Methods.

### Study participants

Study participants were recruited at Helsinki University Hospital (HUH), Helsinki, Finland. Healthy controls were collected from Finnish Red Cross Blood Service (Helsinki, Finland). All participants gave written informed consent before participation. The ethics committee in the HUH approved the study, and it was conducted in accordance with the Declaration of Helsinki. Patients’ clinical characteristics are shown in Supplementary Table 1 as well as assays in which samples were used.

### Cell line

Previously published [20] KAI3 NK cells engineered to express *STAT3* wildtype (*STAT3*^WT^) or activating mutations of *STAT3* (*STAT3*^G618R^ and *STAT3*^Y640F^) were used to validate the functional effects of *STAT3* mutations. Cells were maintained in RPMI 1640 Medium containing 10% FBS, 1% penicillin/streptomycin, 2 mM L-glutamine, and 100 IU/ml recombinant human Interleukin-2. Mycoplasma test was performed using PCR Mycoplasma Test kit (Cat. PK-CA91-1096, PromoCell, Heidelberg, Germany). Cells were authenticated using GenePrint10 (Promega, Madison, WA, USA). The DSMZ-STR, ATCC-STR, JCRB-STR, and ICLC-STR databases were used to compare the results.

### Plasma cytokine measurements

Plasma cytokine measurement was performed using a Proseek Multiplex Inflammation panel by Olink Proteomics (Uppsala, Sweden) [21]. The relative protein levels are expressed as Normalized Protein eXpression (NPX) in 2-log scale. A larger NPX level indicates a higher protein expression in the sample.

### Human primary CD8+ T cells sorting

Mononuclear cells (MNCs) were isolated using a Ficoll gradient centrifuge. Primary CD8+ T cells were sorted by autoMACS® Pro Separator (Miltenyi Biotec, USA) with CD8 MicroBeads (Miltenyi Biotec, Cat. 130-045-201) from isolated MNCs. The final pools contained >95% purified cells.

### RNA sequencing

Total RNA was extracted from PBMCs of T-LGLL patients and healthy controls. RNA-sequencing using Illumina Hiseq 2000 and further bioinformatic processing were performed as previously reported [22].

### siRNA transfection

siRNAs were purchased from Dharmacon (Chicago, IL, USA). Cells were transfected with 50 nmol/L siRNA using Lipofectamine™ 2000 Transfection Reagent (Thermo Fisher Scientific) in 12-well plates according to manufacturer’s protocols. siRNA sequences are described in Supplementary Table 2.

### Drug sensitivity and resistance testing

Drug sensitivity and resistance testing were performed with primary CD8+ T cells of both T-LGLL patients harboring *STAT3*^Y640F^ mutation and healthy controls. Azacitidine (HY-10586, CAS 320-67-2) was purchased from MedChemExpress (Monmouth Junction, NJ, USA). The experiment was performed as previously described [23].

### Data analysis and statistics

Statistical analysis was performed using GraphPad Prism (Version 8.4.3) and R 4.0.3. Comparisons between the two groups were made using unpaired two side t-test and Mann–Whitney *U* test. *P* value < 0.05 was considered as statistically significant.

## Results

### Plasma inflammatory cytokine levels are increased in LGLL patients

**Fig. 1:**
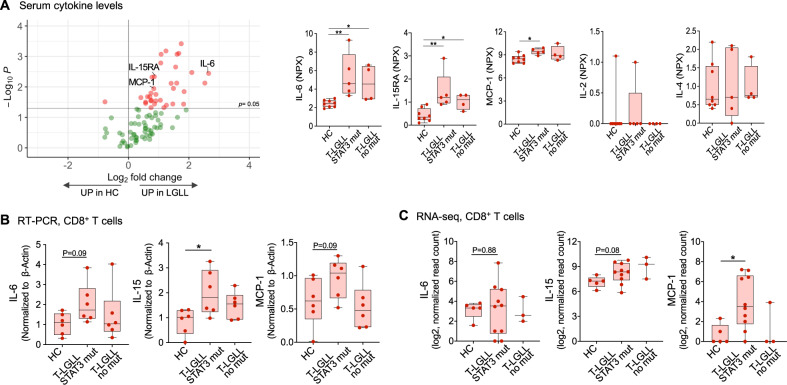
Increased cytokine levels in LGLL patients. **A** Plasma cytokine levels were measured using a Proseek Multiplex Inflammation I immunoassay (Olink Proteomics, Sweden) in CD8+ T cells of healthy controls (HC, *n* = 8), LGLL patients harboring *STAT3* somatic mutation (*n* = 3, *STAT3*^Y640F^; *n* = 1, *STAT3*^D661Y^; *n* = 1, *STAT3*^Y657ins^), or without *STAT3* mutations (*n* = 4). Left, A volcano plot of the normalized protein expression (NPX). The plot shows 91 plasma cytokines differentially expressed between CD8+ T cells from LGLL patients and healthy controls. The red dots indicate significantly higher genes in LGLL patients or healthy controls. Solid line indicates *P* value < 0.05 (Mann–Whitney *U* test). Right, plasma protein NPX level of IL-6, IL-15RA, MCP-1, IL-2, and IL-4. Helthy control vs LGLL with *STAT3* GOF mutations vs LGLL without *STAT3* mutations. **B** Relative mRNA expression of IL-6, IL-15, and MCP-1 were determined using RT-qPCR in the CD8+ T cells of healthy controls (HC), T-LGLL patients harboring *STAT3* somatic mutation (T-LGLL STAT3 mut; *n* = 5, *STAT3*^Y640F^; *n* = 1, *STAT3*^D661Y^), and without *STAT3* mutations (T-LGLL no mut) (*n* = 6 for each group). The sequences of primers were listed in Supplementary Table 4. **C** RNA-seq analysis of *IL-6*, *IL-15,* and *MCP-1* in CD8+ T cells of T-LGLL patients harboring *STAT3* mutations (T-LGLL STAT3 mut; *n* = 5, *STAT3*^Y640F^; *n* = 5, *STAT3*^D661Y^), patients without *STAT3* mutations (T-LGLL no mut, *n* = 3) and healthy controls (HC, *n* = 5). Each dot represents one individual. Data are expressed as mean ± SD, and statistically significant difference was evaluated using Mann–Whitney *U* test. *P < 0.05; **P < 0.01.

We measured plasma cytokine levels using Olink inflammation assay panel [21], including 91 proteins in nine T-LGLL patients (*STAT3*^Y640F^, *STAT3*^D661Y^, *STAT3*^Y657ins^, and without *STAT3* mutations) and eight healthy controls. 34 out of 91 cytokines were higher in T-LGLL patients compared to healthy controls (Fig. 1A, Supplementary Table 3), suggesting broad cytokine upregulation. Specifically, IL-6 and IL-15RA were higher in T-LGLL patients compared to healthy controls irrespective of *STAT3* mutation status (Fig. 1A). Of note, IL-15 was not included in the Olink inflammation panel used (Supplementary Table 3). Monocyte chemoattractant protein 1 (MCP-1) levels were also upregulated in patients with *STAT3* mutations, whereas for example IL-2 and IL-4 levels were similar between patients and healthy controls (Fig. 1A).

We next examined *IL-6*, *IL-15*, and *MCP-1* mRNA expression levels in CD8+ T cells using RT-qPCR. IL-15 expression levels were significantly higher in *STAT3* mutated T-LGLL, whereas only borderline differences in expression levels were observed for IL-6 and MCP-1 (Fig. 1B), suggesting that other cell types contribute to circulating cytokine levels [13]. Similar expression levels were also seen in RNA-sequencing of normal and T-LGLL CD8+ T cells (*STAT3*^Y640F^, *STAT3*^D661Y^, and without *STAT3* mutations) (Fig. 1C, Supplementary Fig. 1).

### Epigenetic regulator levels are linked with *STAT3* mutation status in T-LGLL

**Fig. 2:**
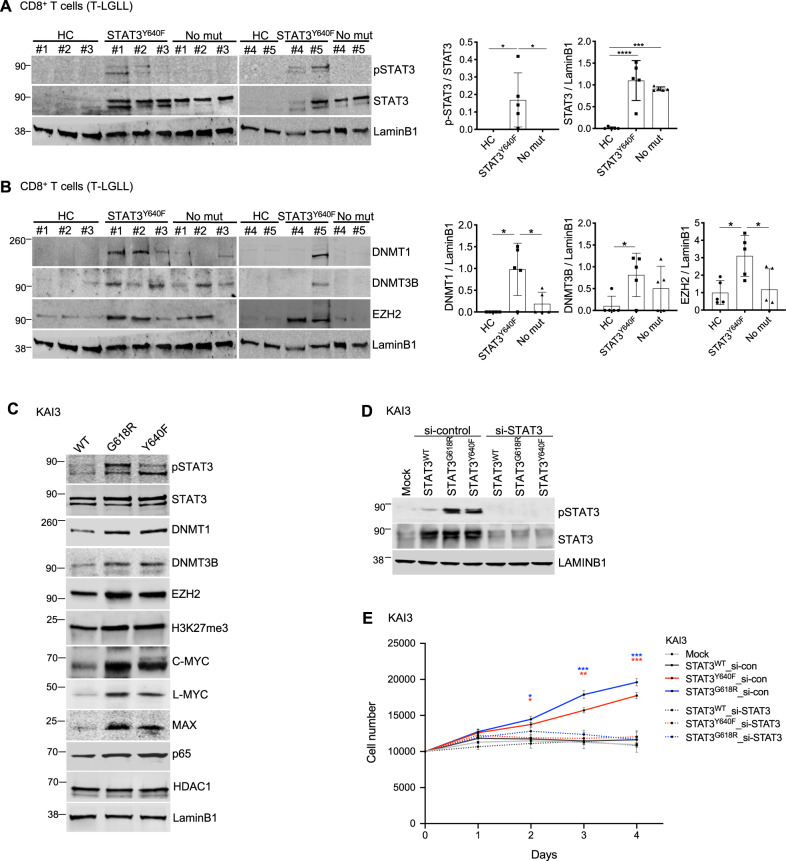
Epigenetic regulator levels are linked with *STAT3* mutation status in LGLL. Western blot analysis of CD8+ T cells from T-LGLL patients. Nuclear fractions from CD8+ T cells of healthy controls (HC, *n* = 5), T-LGLL patients harboring *STAT3*^Y640F^ mutation (STAT3^Y640F^, *n* = 5) and without *STAT3* mutations (No mut, n = 5) were extracted. **A** Left, data are shown as phosphorylated STAT3 (p-STAT3) and STAT3. Right, quantitative presentation of Western blot assays using Image J (Version 2.0.0). Protein levels were first normalized to LAMINB1, a loading control. The p-STAT3 level was normalized by dividing intensity of p-STAT3 by total STAT3. **B** Left, DNMT1, DNMT3B, and EZH2 protein expression levels. Right, quantitative presentation of the protein levels. DNMT1, DNMT3B, and EZH2 protein levels were normalized with LAMINB1. Each dot represents the protein expression level from one individual. Error bar present mean ± SD (*n* = 5 per group), and statistically significant difference was evaluated using Mann–Whitney *U* test. *P < 0.05; ***P < 0.001; ****P < 0.0001. **C** KAI3 NK cells harboring *STAT3*^WT^ (WT), *STAT3*^G618R^ mutation (G618R) and *STAT3*^Y640F^ mutation (Y640F) were serum starved for 12 h followed by nucleus extraction. Protein samples were analyzed via Western blot analysis using the indicated antibodies. Data are representative of three independent experiments. Protein quantification is presented in Supplemental Fig. 3A. **D** KAI3 NK cells (*STAT3*^WT^, *STAT3*^G618R^, and *STAT3*^Y640F^) were transfected with *STAT3-siRNA* (si-STAT3) and *control siRNA* (si-con) for 72 h followed by serum starvation for 12 h Western blot analysis was performed with p-STAT3 and STAT3 specific antibodies. LAMINB1 served as loading control and data are representative of three independent experiments. **E** Cell proliferation assay was peformed using *STAT3* siRNA-mediated knockdown in KAI3 NK cells. KAI3 NK cells stably expressing *STAT3*^WT^ and gain-of-function *STAT3* variants (*STAT3*^G618R^ and *STAT3*^Y640F^) were cultured with *STAT3*-*siRNA* (si-STAT3) and *control siRNA* (si-con) for 48 h The cells (10,000 cells/well) were incubated in RPMI1640 with reduced IL-2 (25IU/ml) with extra si-RNAs for indicated days, and the cell proliferation was measured by DNA fluorescence-based assay. The cell numbers were calculated with a standard curve based on the fluorescence intensity. Error bar present mean ± SD of three independent experiments, and statistically significant difference was evaluated using unpaired *t*-test (STAT3^Y640F^ si-con vs STAT3^Y640F^ si-STAT3, STAT3^G618R^ si-con vs STAT3^G618R^ si-STAT3). *P < 0.05; **P < 0.01; ***P < 0.001.

Using nuclear fractions of CD8+ T cells, we observed high STAT3 phosphorylation levels in T-LGLL patients harboring *STAT3*^Y640F^ mutation, in accordance with previous reports (Fig. 2A) [3, 4, 24]. In addition, total STAT3 levels were significantly increased in T-LGLL patients compared with healthy controls.

To examine whether STAT3 regulates DNMT activity and DNA methylation in T-LGLL, we quantified DNMT protein levels in patient samples. Interestingly, DNMT1 and DNMT3B levels were significantly higher in CD8+ T cells of the LGLL patients with *STAT3*^Y640F^ GOF mutation compared to healthy controls (Fig. 2B). The protein level of EZH2, a histone H3K27 methyltransferase, was also higher in T-LGLL patients, particularly in patients with *STAT3*^Y640F^ mutation. In addition, we observed high level of MYC protein in T-LGLL patients harboring *STAT3*^Y640F^ GOF mutation (Supplementary Fig. 2).

To further investigate whether *STAT3*^Y640F^ mutation can upregulate epigenetic regulators, we used KAI3 NK cells previously engineered to overexpress exogenous *STAT3* wildtype (*STAT3*^WT^) and hyperactive *STAT3* variant (*STAT3*^Y640F^) [20] found in T-LGLL patients [3–5]. We also tested KAI3 cells expressing *STAT3*^G618R^ variant [20] to further examine the effect of *STAT3* GOF mutations. DNMT1 and DNMT3B were significantly upregulated in KAI3 NK cells overexpressing *STAT3* GOF variants compared to cells overexpressing *STAT3*^WT^ (Fig. 2C, Supplementary Fig. 3A). In addition, EZH2 protein level and levels of H3K27me3, the end-product of histone methylation catalyzed by EZH2 as part of the PRC2 complex [25], were increased in cells overexpressing *STAT3* GOF variants (Fig. 2C, Supplementary Fig. 3A). DNMT3A protein was not detected in KAI3 NK cells (Supplementary Fig. 3B). Similar to patient cells, the expression of MYC family proteins, including C-MYC and L-MYC, were increased in KAI3 NK cells overexpressing *STAT3* GOF mutations (Fig. 2C). Further, MYC-associated factor X (MAX) [26] was upregulated in KAI3 NK cells expressing *STAT3* variants (Fig. 2C). The protein level of p65, a subunit of NF-κB reported to be upregulated in MYC-dependent manner upon IL-15 stimulation [17], was also increased in hyperactive *STAT3* KAI3 NK cells (Fig. 2C). Although HDAC1 has been reported to be upregulated by excessive production of IL-15 in T cells [27], we did not find any difference in HDAC1 levels according to the *STAT3* status (Fig. 2C, Supplementary Fig. 3A).

Overexpression of GOF *STAT3* variants have been reported to promote cell proliferation in KAI3 NK cells under limiting IL-2 concentrations [20]. To further validate that this was due to *STAT3* activation, we performed si-RNA mediated STAT3 knockdown in KAI3 NK cells (Fig. 2D). While *STAT3* GOF expression led to increased cell proliferation in KAI3 NK cells, STAT3 knockdown rescued this phenotype (Fig. 2E). We additionally confirmed that STAT3 knockdown attenuated C-MYC (Supplementary Fig. 4), further supporting a link between STAT3 and MYC pathway.

### Cytokines activate STAT3 and epigenetic regulator signaling in healthy CD8+ T cells

**Fig. 3:**
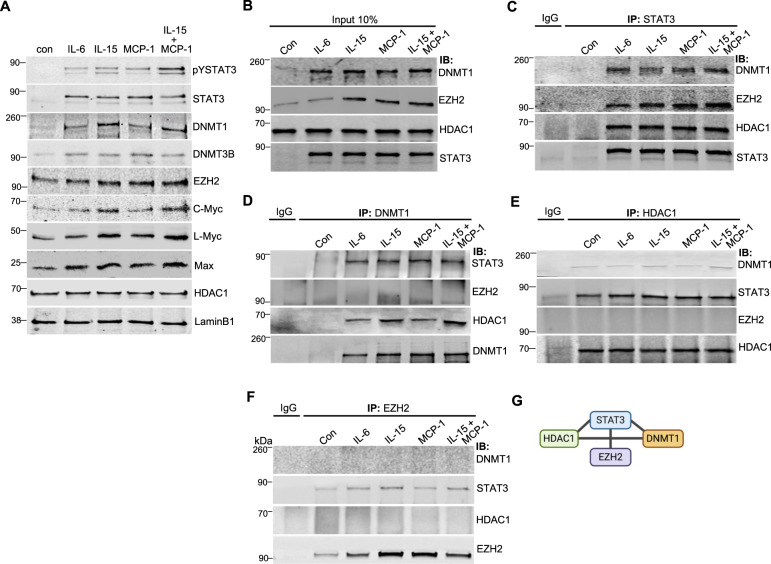
Cytokines activate STAT3 that binds epigenetic regulators in healthy CD8+ T cells. **A** Western blot analysis of CD8+ T cells of healthy controls. Cells were cultured with IL-6, IL-15, MCP-1, IL-15 plus MCP-1 and without cytokine (con) in RPMI1640 medium for 12 h (100 ng/ml for each cytokine). Nuclear fractions of the cells were used to detect the indicated proteins. LAMINB1 was used as loading control. **B**–**F** Physical interaction of STAT3 with DNMT1, EZH2, and HDAC1. CD8+ T cells of healthy controls were cultured with IL-6, IL-15, MCP-1, and IL15 plus MCP-1 in RPMI1640 medium for 12 h to induce STAT3 activation. Co-Immunoprecipitation was performed with the nuclear protein lysates of CD8+ T cells. **B** The nuclear extracts (10%) of the cells were used as input control. Co-IP was performed with antibody against **C** STAT3, **D** DNMT1, **E** HDAC1, and **F** EZH2. Rabbit IgG Isotype control (IgG) was used as a negative control for non-specific binding for Co-IP. Immunoprecipitated complexes were probed using the antibodies as indicated. Data are representative of three independent experiments. **G** Simplified scheme of the complex of STAT3, DNMT1, EZH2, and HDAC1 using BioRender (Toronto, Canada).

To address whether high cytokine levels are sufficient to activate STAT3 and the epigenetic machinery in CD8+ T cells, we isolated primary CD8+ T cells from healthy blood donors. These cells were cultured with IL-6, IL-15, and MCP-1, which were selected based on their higher levels in T-LGLL patients relative to healthy controls (Fig. 1A). Cytokines increased CD8+ T cell proliferation rate after 12 h of stimulation (Supplementary Fig. 5). Using four different cytokine stimulation conditions (IL-6, IL15, MCP-1, IL15 plus MCP-1) for equal time span in saturating cytokine concentrations, we observed that each cytokine increased the phosphorylation of STAT3, as well as total protein levels of STAT3, DNMT1, DNMT3B, EZH2, and MYC family proteins (Fig. 3A, Supplementary Fig. 6A). DNMT3A was not detected (Supplementary Fig. 6B). CD8+ T cells treated with a combination of IL-15 and MCP-1 showed the highest level of p-STAT3 (Fig. 3A), suggesting that in addition to direct effects of *STAT3* mutations, increased levels of IL-6, IL-15, or MCP-1 (Fig. 1A, B) may further hyperactivate STAT3, DNMT1, and MYC signaling in LGLL.

### STAT3 directly binds epigenetic regulator proteins

To understand the connection between STAT3 and epigenetic modifiers, we performed Co-immunoprecipitation (Co-IP) in CD8+ T cells isolated from healthy controls and stimulated with IL-6, IL-15, MCP-1, or IL-15 plus MCP-1. STAT3 was able to bind DNMT1, EZH2, and HDAC1 (Fig. 3B–E). EZH2 directly interacted with STAT3 but not within a higher-order complex including HDAC1 or DNMT1 (Fig. 3F–G). Consistent with findings in stimulated healthy CD8+ T cells, we observed stronger interaction between STAT3 and DNMT1 as well as between STAT3 and HDAC1 in KAI3 NK cells overexpressing GOF *STAT3* compared to *STAT3*^WT^ (Supplementary Fig. 7).

### STAT3 activation is associated with DNA hypermethylation

**Fig. 4:**
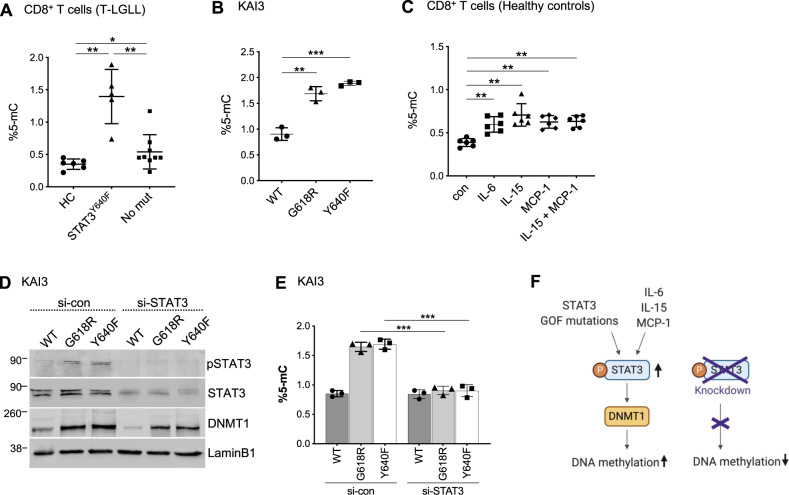
Quantification of global DNA methylation level and the effect of STAT3 depletion. **A** Global DNA methylation levels in CD8+ T cells of healthy controls (HC, *n* = 6), T-LGLL patients harboring *STAT3*^Y640F^ mutation (STAT3^Y640F^, *n* = 5), and T-LGLL patients without *STAT3* mutations (No mut, *n* = 9). %5-mC were evaluated by fluorescence according to manufacturer’s instruction. **B** KAI3 NK cells stably expressing *STAT3*^WT^ (WT), *STAT3*^G618R^ (G618R) and *STAT3*^Y640F^ (Y640F) were cultured without serum for 12 h followed by genomic DNA extraction and global DNA methylation measurement. **C** Global DNA methylation levels in CD8+ T cells of healthy controls stimulated with IL-6, IL-15, and/or MCP-1 for 12 h were measured (*n* = 6 for each group). Error bar present mean ± SD and statistically significant difference was evaluated using Mann–Whitney *U* test. *P < 0.05; **P < 0.001; ***P < 0.001. **D** Protein expression in KAI3 NK cells transfected with *STAT3-siRNA* (si-STAT3) and *control siRNA* (si-con) for 72 h in RPMI1640 with reduced IL-2 (25IU/ml) followed by serum starvation for 12 h. Protein extracts from siRNA-treated cells were subjected to Western blot analysis with p-STAT3, STAT3, and DNMT1 specific antibodies. LAMINB1 served as loading control and data are representative of three independent experiments. **E** Global methylation level in KAI3 NK cells transfected with *STAT3-siRNA* and con-siRNA for 72 h in RPMI1640 with reduced IL-2 (25IU/ml). Error bar present mean ± SD (*n* = 3 per group), and statistically significant difference was evaluated using Mann–Whitney *U* test. Error bar present mean ± SD and statistically significant difference was evaluated using Mann–Whitney *U* test. ***P < 0.001. **F** Simplified scheme of the *STAT3* GOF mutations. Pathway components discovered to be upregulated with functional assays are marked with arrows. The schematic was made using BioRender (Toronto, Canada).

We next quantified global DNA methylation in healthy and T-LGLL CD8+ T cells as well as in KAI3 NK cells. CD8+ T cells from T-LGLL patients harboring S*TAT3*^Y640F^ mutation had the highest global DNA methylation levels (median = 1.44) compared to cells from T-LGLL patients without *STAT3* mutations (median = 0.45) or from healthy controls (median = 0.365) (Fig. 4A). Similarly, the methylation levels were significantly higher in *STAT3*^Y640F^ (median = 1.90) and *STAT3*^G618R^ (median = 1.72) cells compared to *STAT3*^WT^ KAI3 NK cells (median = 0.86) (Fig. 4B). Furthermore, stimulation of healthy control CD8+ T cells with cytokines led to higher global DNA methylation levels (Fig. 4C). IL-15 (median = 0.70) showed the highest impact on upregulating DNA methylation among the cytokine conditions used (median: IL-6 = 0.64, MCP-1 = 0.61, IL-15+MCP-1 = 0.68, without treatment = 0.40).

We then evaluated whether STAT3 inhibition can suppress DNA methylation using KAI3 NK cells and a small interfering RNA (siRNA) targeting STAT3. Consistent with results from a prior study [28], DNMT1 levels decreased in response to STAT3 inhibition (Fig. 4D). Global methylation levels decreased with *STAT3* siRNA in KAI3 NK cells overexpressing *STAT3* mutants, but not in *STAT3*^WT^ cells (Fig. 4E), suggesting that the upregulated DNA methylation is caused by STAT3 activity and DNMT1 (Fig. 4E, F).

### Increased ROS levels associated with *STAT3* activation and cytokine stimulation

**Fig. 5:**
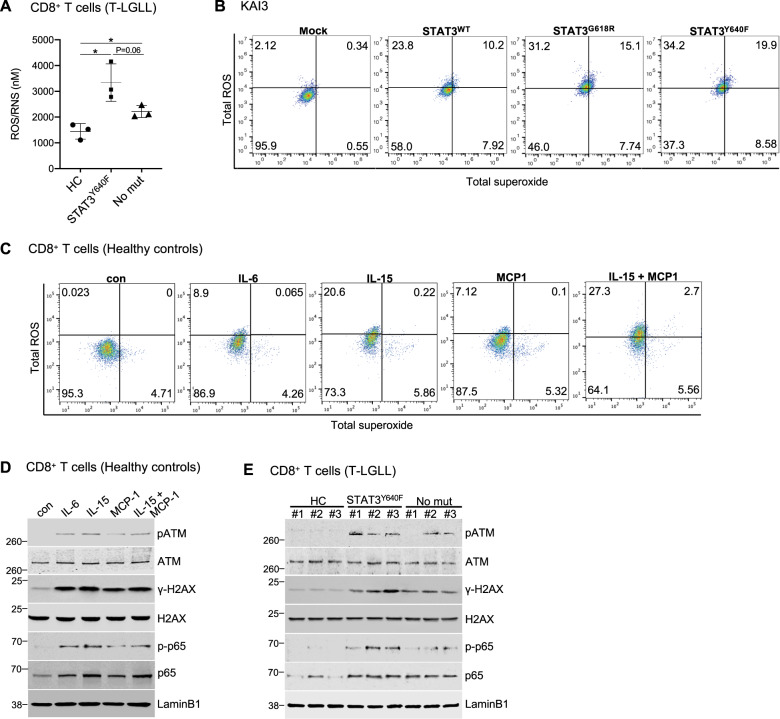
Increased oxidative stress is related to STAT3 mutations. **A** The level of ROS/RNS in CD8+ T cells of T-LGLL patients harboring *STAT3*^Y640F^ mutation (STAT3^Y640F^) and without *STAT3* mutations (No mut) as well as healthy controls (HC) (*n* = 3). Error bars expressed as mean ± SD, and statistically significant difference was evaluated using unpaired *t*-test. *P < 0.05. **B** Flow cytometry analysis to examine the expression of total ROS and superoxide in KAI3 NK cells harboring *STAT3*^WT^, STAT3^G618R^, *STAT3*^Y640F^ mutation and control KAI3 NK cells (Mock). Cells were cultured in RPMI1640 with reduced IL-2 (25IU/ml) for 72 h Then, the cells were labeled with ROS-fluorescent dye (Ex/Em= 650/675 nm) and superoxide-fluorescent dye (Ex/Em = 520/605 nm), and then analyzed using flow cytometer. KAI3 NK cells expressing empty vector (Mock) were used as a control. **C** Flow cytometry analysis to examine the expression of total ROS and superoxide in untreated CD8+ T cells (con) and those incubated with IL-6, IL-15, MCP-1, or IL15 plus MCP-1. CD8+ T cells were incubated with the indicated cytokines (100 ng/ml) for 12 h in RPMI1640. Cells were labeled with ROS-fluorescent dye and superoxide-fluorescent dye, and then analyzed using flow cytometer. **D** p-ATM, ATM, γH2AX, H2AX, p65 and p-p65 protein expression in healthy CD8+ T cells stimulated with IL-6, IL-15, and/or MCP-1 for 12 h. Quantification is presented in Supplementary Fig. 8A. **E** p-ATM, ATM, γH2AX, H2AX, p65, and p-p65 protein expression in CD8+ T cells of T-LGLL patients harboring *STAT3*^Y640F^ mutation (STAT3^Y640F^) and without *STAT3* mutations (No mut) as well as healthy controls (HC). Quantification is presented in Supplementary Fig. 8B.

Since a link between DNA methylation and oxidative stress has been suggested [29], we measured ROS levels in CD8+ T cells from T-LGLL patients using an in vitro ROS/RNS (reactive nitrogen species) assay. ROS/RNS levels were significantly higher in T-LGLL patients harboring *STAT3*^Y640F^ mutation (median = 3070) compared with both healthy controls (median = 1528) and the patients without *STAT3* mutations (median = 2117) (Fig. 5A). Interestingly, KAI3 NK cells overexpressing *STAT3* (either wildtype or *STAT3* GOF) showed increased levels of both ROS and superoxide compared to the control KAI3 cells. Moreover, ROS levels in cells carrying *STAT3* GOF mutations were higher compared to KAI3 NK cells expressing *STAT3*^WT^ (Fig. 5B).

We investigated whether cytokines can also promote ROS production in healthy CD8+ T cells. In line with our primary LGLL patient data, total ROS was significantly increased from 0.023% (control) to 9.6% (IL-6), 20.8% (IL-15), 7.2% (MCP-1), and 30.0% (IL-15 plus MCP1) (Fig. 5C). Superoxide levels were also increased in response to IL-15 (6.1%), MCP-1 (5.4%), and IL-15 plus MCP-1 (8.3%) compared to control cells (4.7%), whereas IL-6 (4.3%) had no effect on the production of superoxide. Cytokine stimulation also increased p-ATM and γH2AX in CD8+ T cells (Fig. 5D, Supplementary Fig. 8A), indicating concomitant activation of the DNA damage response pathway [30]. Ser-276 phosphorylation of NF-κB p65 was also noted in cytokine-stimulated CD8+ T cells (Fig. 5D, Supplementary Fig. 8A), suggesting ROS-dependent NF-κB signaling activation [31]. In addition, these proteins were upregulated in CD8+ T cells of T-LGLL patients harboring *STAT3* GOF mutation (Fig. 5E, Supplementary Fig. 8B). Taken together, these results suggest that *STAT3* driver mutations and higher p-STAT3 activation are associated with higher ROS production and activation of the DNA damage response and NF-κB pathways, potentially contributing to LGLL phenotype.

### Azacitidine abrogates STAT3 activation, DNA methylation, and ROS production

**Fig. 6:**
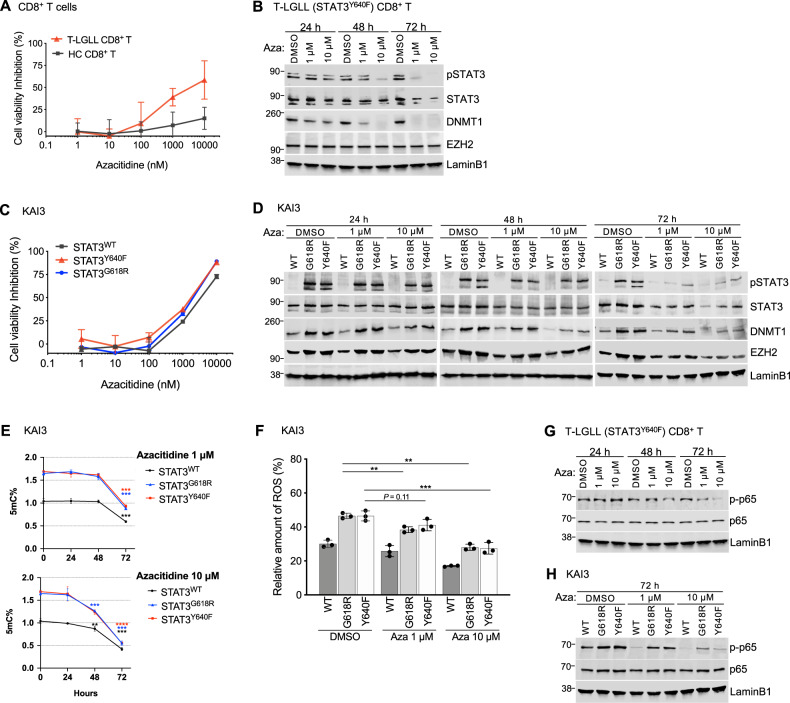
Azacitidine abrogates DNA hypermethylation and STAT3 activation in KAI3 NK cells. **A** Drug sensitivity testing was performed on CD8+ T cells from T-LGLL patients harboring *STAT3*^Y640F^ mutation (STAT3^Y640F^, *n* = 3) and healthy controls (*n* = 3). Cell viability inhibition by correlation of drug sensitivity score (DSS) was measured with CellTiter-Glo2.0 (Promega, USA). DSS is a quantitative measurement of a drug response based on the area under the curve (AUC) with further normalization. Higher DSS denote better killing activity. Dose-response curve of LGLL patients (red line) and healthy controls (gray line) for azacitidine. **B** CD8+ T cells from a T-LGLL patient harboring *STAT3*^Y640F^ mutation were cultured in the presence of azacitidine (1 or 10 μM) or absence (DMSO alone) for 24, 48, and 72 h. Cell lysates were immunoblotted with antibodies against p-STAT3, STAT3, DNMT1, and EZH2. LAMINB1, a loading control. Data are representative of three T-LGLL patients harboring *STAT3*^Y640F^ and quantification is presented in Supplementary Fig. 9. **C** Dose-response curve of KAI3 NK cells stably expressing *STAT3*^WT^ (gray line), *STAT3*^G618R^ (blue line), *STAT3*^Y640F^ (red line) for azacitidine (*n* = 2 per group). DSS scores are presented in Supplementary Table 5. **D** KAI3 NK cells stably expressing *STAT3*^WT^, *STAT3*^G618R^ and *STAT3*^Y640F^ were cultured in the presence of azacitidine (1 or 10 μM) or absence (DMSO alone) for 24, 48, and 72 h in RPMI1640 with reduced IL-2 (25IU/ml). Cell lysates were immunoblotted with antibodies against p-STAT3, STAT3, DNMT1, and EZH2. LAMINB1, a loading control. Data are representative of two independent experiments. Quantification of the proteins is presented in Supplementary Fig. 10. **E** Global DNA methylation levels in KAI3 NK cells of *STAT3*^WT^, *STAT3*^G618R^ and *STAT3*^Y640F^ (for each group, *n* = 3) treated with 1 μM of azacitidine (upper) and 10 μM of azicitidine (lower) at 24, 48, and 72 h in RPMI1640 with reduced IL-2 (25IU/ml). %5-mC were evaluated by fluorescence according to manufacturer’s instruction. Error bar present mean ± SD (*n* = 3 per group), and statistically significant difference was evaluated using Mann–Whitney *U* test. **P < 0.01; ***P < 0.001; ****P < 0.0001. **F** ROS expression in KAI3 NK cells stably expressing *STAT3*^WT^, *STAT3*^G618R^, and *STAT3*^Y640F^. Cells were cultured in the presence of azacitidine (1 or 10 μM) or absence (DMSO alone) for 72 h in RPMI1640 with reduced IL-2 (25IU/ml). Cells were labeled with ROS-fluorescent dye then analyzed using flow cytometer. Error bars expressed as mean ± SD, and statistically significant difference was evaluated using unpaired T-test. **P < 0.01; ***P < 0.001. The representative of three independent experiments is presented in Supplementary Fig. 12A. **G** p-p65 and p65 protein expression level from azacitidine (1 or 10 μM) treated CD8+ T cells of T-LGLL patient harboring *STAT3*^Y640F^ mutation. Data are representative of three T-LGLL patients harboring *STAT3*^Y640F^. **H** p-p65 and p65 protein expression level from azacitidine (1 or 10 μM) treated KAI3 NK cells stably expressing *STAT3*^WT^, *STAT3*^G618R^, and *STAT3*^Y640F^. Data are representative of two independent experiments.

To explore whether DNA hypermethylation associated with STAT3 activation could be therapeutically targeted, we evaluated the effects of azacitidine, a hypomethylating agent widely used to treat myeloid malignancies. Ex vivo drug sensitivity testing showed that T-LGLL CD8+ T cells were more sensitive to azacitidine at higher concentrations compared with CD8+ T cells from healthy controls (Fig. 6A). Next, we investigated whether azacitidine decreases the protein levels of STAT3, p-STAT3, DNMT1, and EZH2. CD8+ T cells of a T-LGLL patient harboring *STAT3*^Y640F^ mutation were cultured in the presence or absence of azacitidine (1 and 10 μM) for 24, 48, and 72 h. Azacitidine inhibited p-STAT3, total STAT3, and DNMT1 in dose- and time-dependent manner (Fig. 6B, Supplementary Fig. 9). EZH2 levels were not affected by azacitidine. To validate these results, we treated KAI3 NK cells with azacitidine. While no statistically significant differences in cell viability were observed between cells overexpressing *STAT3*^WT^ or GOF *STAT3* variants (Fig. 6C), protein levels of pSTAT3, total STAT3, and DNMT1 were decreased with azacitidine treatment (Fig. 6D), consistent with results observed in CD8+ T cells. EZH2 level was also decreased after 72 h of 10 μM of azacitidine treatment in KAI3 NK cells (Fig. 6D, Supplementary Fig. 10).

In line with the hypomethylating mechanism of action of azacitidine, KAI3 NK cells showed decreased global methylation levels in time and dose-dependent manner after azacitidine treatment (Fig. 6E, Supplementary Fig. 11). ROS levels were decreased in KAI3 NK cells expressing either *STAT3*^WT^ and *STAT3* GOF variants (Fig. 6F, Supplementary Fig. 12A). In addition, azacitidine reduced Ser-276 phosphorylation of p65 in CD8+ T cells of LGLL patients (Fig. 6G) and KAI3 NK cells (Fig. 6H). Given that ROS can stimulate overall tyrosine phosphorylation-dependent signaling [32], we additionally assessed the effect of azacitidine on total phosphotyrosine levels in KAI3 NK cells. Azacitidine significantly reduced whole cell tyrosine-phosphorylated proteins (25–260 kDa), including p-STAT3 (Supplementary Fig. 12B). Together, these results suggest that DNMT inhibitors can influence epigenetic programming and ROS production in STAT3 activation-dependent manner, with potential clinical applicability in LGLL.

### Azacitidine inhibits STAT3 activation by SHP1 restoration

**Fig. 7:**
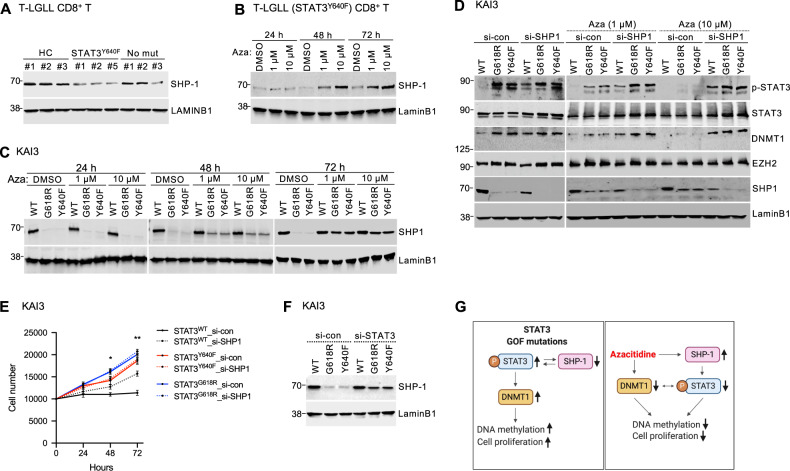
Azacitidine restores SHP-1 by decresing STAT3 activation. **A** SHP1 protein levels at baseline in CD8+ T cells of healthy controls (HC, *n* = 3), T-LGLL patients harboring *STAT3*^Y640F^ mutation (STAT3^Y640F^, *n* = 3) and T-LGLL patient without *STAT3* mutations (No mut, *n* = 3). Quantification is presented in Supplementary Fig. 13. **B** SHP1 protein levels in CD8+ T cells of a T-LGLL patient harboring *STAT3*^Y640F^ mutation treated with azacitidine (1 or 10 μM) for 24, 48, and 72 h Data are representative of three T-LGLL patients harboring *STAT3*^*Y640F*^ and quantification is presented in Supplementary Fig. 14. **C** SHP1 expression in KAI3 NK cells (*STAT3*^WT^, *STAT3*^G618R^, and *STAT3*^Y640F^) treated with azacitidine (1 or 10 μM) for 24, 48, and 72 h in RPMI1640 with reduced IL-2 (25IU/ml) followed by Western blot assay. **D** KAI3 NK cells stably expressing *STAT3*^WT^, *STAT3*^G618R^, and *STAT3*^Y640F^ were treated with *SHP1*-*siRNA* (si-SHP1) and *control siRNA* (si-con) for 72 h followed by azacitidine treatment for 72 h in RPMI1640 including reduced amount of IL-2 (25IU/ml). Western blot was performed with the use of anti-pSTAT3, anti-STAT3, anti-DNMT1, anti-SHP1, and anti-LaminB1 antibodies. **E** KAI3 NK cells stably expressing *STAT3*^WT^ and GOF *STAT3* variants (*STAT3*^G618R^ and *STAT3*^Y640F^) were cultured with *SHP1*-*siRNA* (si-SHP1) and *control siRNA* (si-con) for 48 h. Next, siRNAs transfected cells (10,000 cells/well) were cultured in RPMI1640 with reduced IL-2 (25IU/ml) with extra siRNAs for 72 h, and the cell proliferation was measured by DNA fluorescence-based assay. Cell numbers were calculated with a standard curve based on the fluorescence intensity. Error bar present mean ± SD of three independent experiments, and statistically significant difference was evaluated using unpaired t-test (*STAT3*^WT^ si-con vs *STAT3*^WT^ si-SHP1). *P < 0.05; **P < 0.01. **F** SHP1 expression in KAI3 NK cells transfected with *STAT3-siRNA* (si-STAT3) and *control siRNA* (si-con) for 72 h. The cells were serum starved for 12 h followed by Western blot assay. **G** Simplified scheme of the effect of azacitidine. Pathway components discovered to be upregulated or downregulated with functional assays are marked with arrows. The scheme was made using BioRender (Toronto, Canada).

Previous studies have shown that SHP1 negatively regulates JAK/STAT3 signaling pathway [33, 34]. Basal expression of SHP1 was lower in CD8+ T cells of T-LGLL patients harboring *STAT3*^Y640F^ mutations compared to both healthy controls and T-LGLL patients without *STAT3* mutations (Fig. 7A, Supplementary Fig. 13). Furthermore, azacitidine treatment led to increased SHP1 levels in CD8+ T cells isolated from T-LGLL patients (Fig. 7B, Supplementary Fig. 14). SHP1 levels were lower in KAI3 NK cells overexpressing GOF *STAT3* mutations compared to *STAT3*^WT^ cells at baseline; however, azacitidine treatment increased SHP1 levels in GOF *STAT3* cells (Fig. 7C). Next, KAI3 NK cells were transfected with control and *SHP1* siRNA for 48 h followed by azacitidine treatment. Knockdown of SHP1 upregulated p-STAT3 and DNMT1 in *STAT3*^WT^ cells at baseline (Fig. 7D). In addition, SHP1 knockdown attenuated p-STAT3 and DNMT1 downregulation during azacitidine treatment in KAI3 NK cells. SHP1 knockdown promoted cell proliferation in *STAT3*^WT^ but not in GOF *STAT3* cells, providing further support for the link between STAT3 upregulation and cell proliferation (Fig. 7E). We also confirmed that siRNA-mediated STAT3 knockdown restored SHP1 expression (Fig. 7F). Together, these findings suggest that SHP1 controls STAT3 activity and inhibition of STAT3 during azacitidine treatment (Fig. 7G).

## Discussion

Activating *STAT3* somatic mutations can be found both in patients with T-cell LGLL (40-50% of cases with CD8 + CD3 + phenotype) and in CLPD-NK (20-30% of patients) [3, 6]. *STAT3* is also mutated in other T and NK cell neoplasias such as mature T cell lymphomas and aggressive NK cell leukemias [35–38]. Interestingly, germline *STAT3* GOF variants have also been associated with early LGL lymphoproliferation [39, 40]. Furthermore, a role for JAK/STAT activation in LGLL pathogenesis even in patients without *STAT3* mutations has been suggested [1, 6, 12, 13]. Nevertheless, the consequences of *STAT3* activation in pathological T- and NK-cell proliferations remain elusive. In our study, we show that in primary LGLL cells GOF *STAT3* mutations alter autoregulation of p-STAT3 and drive changes in epigenetic regulator levels, global DNA hypermethylation, and ROS production. In addition to LGLL patient cells, we used KAI NK cells and cytokine-stimulated CD8+ T cells to validate the consequences of STAT3 pathway activation although it needs to be taken into account that due to gain of function *STAT3* mutations, the STAT3 activation in T-LGLL is chronic compared to transient changes induced by cytokine stimulation. Our results were well recapitulated in these models, and we observed higher-order protein complex interactions between STAT3 and EZH2, HDAC1, and DNMT1 in both healthy CD8+ T cells with proinflammatory cytokine treatment and KAI3 NK cells overexpressing GOF *STAT3* variants, indicating a direct link between STAT3 and epigenetic regulation. Azacitidine, a hypomethylating agent, rescued these phenotypes in SHP1 dependent manner. Together, our results suggest a central role for STAT3 and epigenetic changes to drive LGLL pathogenesis, and provide rationale for testing hypomethylating agents in the treatment of LGLL patients resistant to standard therapies.

Prior studies have shown elevated levels of circulating inflammatory cytokines in LGLL [13, 41–43]. The exact mechanism and dominance of certain cytokines versus complex action of several cytokines remains largely enigmatic. We confirmed increased cytokine levels in T-LGLL patients compared to healthy controls. Increased global DNA methylation pattern was found both in CD8 + LGLL cells and in response to stimulation with IL-6, IL-15, and MCP-1 in healthy CD8+ T cells. These global epigenetic changes may partly confer a selection pressure on T cell clones, facilitating clonal expansion after cytokine stimulation. While the increased total methylation pattern in response to cytokine stimulation in CD8+ T cells was confirmed in the fluorescence-based assay, DNA methylation sequencing analyses are warranted to explore the methylated genomic sites in detail. In addition, further studies using primary LGLL cells could link alterations in cytokine profiles and global DNA methylation with key gene expression profiles.

We observed higher levels of circulating IL-15RA in T-LGLL patients samples, and IL-15 promoted epigenetic changes in healthy CD8+ T cells. IL-15-producing macrophages and dendritic cells have been reported to cross-represent IL-15 to promote proliferation and cytotoxicity in LGL [19]. IL-15 can initiate LGLL in transgenic mice via the induction of MYC, p65, and HDAC1 with DNMT3B overexpression and DNA hypermethylation [17]. In addition, IL-15 can maintain the survival of leukemic clones in T-LGL and other T malignancies [44]. We also observed the activation of these proteins in healthy CD8+ T cells stimulated with cytokines, and validated these findings in KAI3 NK cells overexpressing GOF *STAT3* mutations, indicating that enhanced p-STAT3 may promote LGLL pathogenesis in part via induction of these oncogenic pathways by cytokine stimuli.

STAT3 interacts with many key epigenetic regulators and other transcription factors, coactivators, and corepressors to reprogram transcription [45–47]. DNMT1 is closely associated with STAT3 signaling in malignant T cells [48], and our results suggest that STAT3 directly binds DNMT1, EZH2, and HDAC1 to regulate DNA methylation. We showed that STAT3 interacts with DNMT1 and HDAC1 both in CD8+ T cells and KAI3 NK cells, which is in line with previous studies showing STAT3-DNMT1-HDAC1 complex in lymphoma cells [49]. These complexes may contribute to the link between p-STAT3 activation and global DNA methylation.

ROS levels were higher in CD8+ T cells of LGLL patients compared to healthy controls, which is consistent with previous studies showing increased level of ROS in hematological malignancies [50, 51]. KAI3 NK cells expressing *STAT3* GOF variants had higher levels of ROS compared to cells overexpressing *STAT3*^WT^, suggesting that ROS is associated with STAT3 activation. We also observed increased ROS levels after 12-hour cytokine stimulation in healthy CD8+ T cells. Furthermore, we found increased activation of both DNA damage and NF-κB pathways in *STAT3* mutated CD8+ T cells of LGLL patients and healthy CD8+ T cells after cytokine stimulation. Rapidly accumulating DNA damage upon stimulation of CD8+ T cells may lead to increased mutagenesis and clonal selection, while chronic elevation in ROS and DNA damage may be markers of increased clonal turnover in T-LGLL patients. Given that inflammatory cytokines are commonly associated with chronic inflammation and ROS production [52], our results suggest that STAT3 activation, high ROS levels, DNA damage, and NF-kB signaling are associated with LGLL phenotype.

While current LGLL treatment protocols rely on immunosuppressive therapies, targeted therapies such as NF-κB inhibitors are under clinical investigations [1]. We demonstrated that DNMT inhibition can potently inhibit the STAT3-DNA methylation pathway in both CD8+ T cells of LGLL patients and KAI3 NK cells expressing activating *STAT3* variants. Azacitidine, a therapeutic agent with DNA hypomethylating activity as the primary mechanism of action [53], inhibited ROS dependent NF-κB activation as well as STAT3 activation via restoration of SHP1, which is in line with a previous study using CD34 cells from AML patients [54] and anaplastic large cell lymphoma cells [55]. The negative correlation between SHP1 and p-STAT3 after azacitidine treatment in KAI3 NK cells is consistent with previous studies in other cell types [33, 56, 57]. SHP1 is primarily expressed in hematopoietic cells and is associated with DNA methylation and leukemogenesis [58]. As STAT3 can promote *SHP1* gene silencing by forming complexes with DNMT1 and HDAC1 [49], the observed restoration of SHP1 expression by azacitidine might be explained by downregulated STAT3 activation. Conversely, siRNA targeting SHP1 was sufficient to abrogate the inhibitory effect of azacitidine on STAT3, suggesting that SHP1 is required for STAT3 inhibition by azacitidine treatment. The effect of azacitidine treatment on upregulating SHP-1 was confirmed in CD8+ T cells collected from a LGLL patient harboring *STAT3*^Y640F^ mutation, suggesting that azacitidine may be used to target *STAT3* mutated cells in LGLL patients. The treatment of LGL cells with demethylating drugs has also been shown to lead to upregulation of SOCS3 providing additional mechanism for observed STAT3 downregulation [13].

In conclusion, our results indicate that proinflammatory cytokine stimulation and *STAT3* GOF mutations lead to epigenetic reprogramming, increased global DNA methylation, increased ROS production, and enhanced proliferation that may drive LGLL disease pathogenesis. The activity of this axis could be partially rescued by DNMT inhibition, providing novel targeted treatment options for LGLL.

## Supplementary information

Supplementary material
