# Supplementary material for: STAT3 activation in large granular lymphocyte leukemia is associated with cytokine signaling and DNA hypermethylation
Source: Leukemia. 2021 Jun 1;35(12):3430–43. doi: 10.1038/s41375-021-01296-0 (PMC8632689; doi:10.1038/s41375-021-01296-0)
Supplement: Supplementary file 1 — Supplementary material [file 41375_2021_1296_MOESM1_ESM.pdf]

# Supplementary Information

# Supplementary methods

### DNA extraction and amplicon sequencing

Genomic DNA was isolated using NucleoSpin® Tissue Kit (MACHEREY-NAGEL, Cat. 740952) with proteinase K digestion and RNase treatment. Genomic DNA was quantified with QubitTM dsDNA HS Assay kit (Thermo Fisher Scientific, Cat. Q32851).

*STAT3* mutations were validated by *STAT3* targeted amplicon sequencing. Amplicons were amplified with two-step PCR as previously described using DNA Engine Tetrad 2 (Bio-Rad, USA) [1]. Sequencing reads alignment was done with Bowtie2, and GATK IndelRealigner was used for local realignment near indel. The variant was called if variant base frequency was 0.5% of all reads covering a given a position. All variants with the base quality frequency ratio (ratio of number of variant calls/numbers of all bases and quality sum of variant calls / quality sum of all bases at the position) ≥ 0.9 were considered as true somatic variants [2].

### Quantitative reverse transcription PCR (RT-qPCR)

Total RNA was isolated from human primary CD8+ T cells using RNeasy Mini Kit (Cat. 74104, Qiagen, Hilden, Germany). With 1µg of RNA the reverse transcription was performed using QuantiNova Reverse Transcription Kit (Cat. 205411, Qiagen) according to manufacturer's instruction. PowerUp™ SYBR® Green Master Mix (Cat. A25742, Applied Biosystem, Foster City, CA, USA) was used to quantify the gene expression. RT-qPCR was performed using QuantStudio 6 Flex Real-Time PCR System (Applied Biosystems). Beta actin (*ACTB*) was used to normalize gene expression level and the relative quantitation of gene expression was analyzed using comparative cycle threshold (ΔΔCT) approach. The sequences of primers were listed in Supplementary Table 4.

**Cell proliferation assay**

Human primary CD8+ T cells were incubated with IL-6, IL-15, MCP-1, or IL-15 plus MCP-1 (100 ng/ml) in RPMI1640 for up to 72 hours. Cells were washed with cold PBS followed by fixation in ice cold 70% EtOH at 4°C. Cells were centrifuged (200 g, 4°C for 10 mins), and washed with cold 1 X PBS. Rnase A (Cat. EN0531, Thermo Fisher Scientific) was treated (0.5 mg/ml) to stain only DNA (at 37°C for 15 min), and then propidium iodide (Cat. P3566, Thermo Fisher Scientific) was added (10 μg/ml). The percentage of cells in S and G2/M phases of the cell cycle were analyzed with BD Accuri™ C6 Plus and BD AccuriTM analysis software (BD Biosciences, San Jose, CA, USA).

CyQUANT™ Cell Proliferation Assay (Cat. C35011, Invitrogen, Carlsbad, CA, USA) was used for KAI3 NK cells. Briefly, 100 μL of the cell suspension was plated in 96 well plate. At indicated time point, 100 μL of detection reagent combined with background suppressor and nucleic acid stain dye was added to the cells. After incubation for 1 h at 37°C, the fluorescence was measured with FITC filter. Cell numbers were calculated using the standard curve according to the manufacturer's instruction.

**Western blot analysis**

The nuclear extraction was performed using NE-PER™ Nuclear and Cytoplasmic Extraction Reagents (Thermo Fisher Scientific, Cat. 78833) according to the manufacturer's instruction. Total protein extraction was obtained using RIPA buffer (Thermo Fisher Scientific, Cat. 89900) with 1 X protease and phosphatase inhibitor (Thermo Fisher Scientific, Cat. 78440) on ice. Protein concentration measurement and Western blot were performed as previously reported.[1]

**Antibodies**

Primary antibodies against pSTAT3-Tyr705 (Cat. 9145), STAT3 (Cat. 12640), EZH2 (Cat. 5246), MAX (Cat. 4739), NFkB-p65 (Cat. 8242), Rabbit IgG Isotype control (Cat. 3900S), ACTB (Cat. 3700), DNMT3B (Cat. 67259), phospho-ATM (Cat. 5883), ATM (Cat. 2873), γH2AX (Cat. 9718), H2AX (Cat. 7631), Tri-Methyl-Histone H3 (Cat. 9733T) were purchased from Cell signaling

Technology (MA, USA). DNMT1 (Cat. ab92314), HDAC1 (Cat. ab19845), c-MYC (Cat. ab32072), LAMINB1 (Cat. ab133741) were purchased from Abcam (Cambridge, UK). L-MYC (R&D Systems, Cat. AF4050), SHP-1 (Santa Cruz Biotechnology, Cat. Sc-7289) and phosphotyrosine-4G10 (Merck, Cat. 05-321X) were used. Phospho-p65 (Ser276, Cat. NB100-82086) was purchased from Novus Biologicals (CO, USA). IRDye® 800CW Goat anti-Rabbit IgG (Li-COR Biosciences, Cat. 926-32211), IRDye® 680RD Goat anti-Mouse IgG, Anti-mouse IgG (CST, Cat. 7076P2), Anti-rabbit IgG (CST, Cat. 7074), and Anti-Goat IgG (R&D Systems, Cat. AF7237) were used as secondary antibodies.

**Quantification of methylated DNA**

Genomic DNA were isolated using NucleoSpin® Tissue Kit (Cat. 740952, MACHEREY-NAGEL, Düren, Germany). DNA concentration was measured with Qubit® 2.0 Fluorometer (Thermo Fisher Scientific), and 100 ng of DNA were used per reaction according to the instruction of Methylated DNA Quantification Kit (ab117129, Abcam). Relative fluorescence units (RFU) were read on Pherastar FS microplate reader (BMG Labtech, Mölndal, Sweden) at Ex/Em = 530/590 nm. The % 5-methylcytosine (5-mC) was calculated by following the formulae in manufacturer's instruction.

**Measurement of reactive oxygen species (ROS)**

Human primary CD8+ T cells and KAI3 NK cells were seeded in 12-well plates (0.5 x $10^6$/well). CD8+ T cells were incubated in RPMI1640 medium for 12 h with IL-6, IL-15, and/or MCP-1 (100 ng/ml). KAI3 NK cells were cultured in RPMI1640 with IL-2 (25IU/ml). Samples were prepared with ROS-fluorescent dye (Cat. ab186029, Abcam) and superoxide-fluorescent dye (Cat. Ab219943, Abcam) according to manufacturer's protocol. ROS/Superoxide expression was measured by BD Accuri™ C6 Plus Flow Cytometer (BD Biosciences, USA) and BD AccuriTM analysis software (BD Biosciences). We used Oxiselect™ in vitro ROS/RNS assay kit (Cell Biolabs, Cat. STA-347) for T-LGLL patients' samples. The presence of ROS from CD8+ T cells of T-LGLL patients and healthy controls were determined according to the manufacturer's

instruction. Briefly, CD8+ T cells (5 x $10^6$) were sonicated in 200 µl of icd-cold PBS with 0.5% of NP40 and centrifuged at 10,000 g for 5 min followed by storage at -80 ˚C until use. 50 µl of the supernatant were incubated with Catalyst to accelerate the oxidative reaction. DCHF probe solution which can react with ROS and RNS species was added and incubated at room temperature for 30 minutes. Fluorescences were measured by Pherastar FS microplate reader (BMG Labtech, Mölndal, Sweden) at Ex/Em = 480/530 nm. The hydrogen peroxide standard curve was used to quantify the free radical content in CD8+ T cells.

**Co-immunoprecipitation**

The nuclear lysates were pre-cleared with PureProteome™ Protein A/G Mix Magnetic Beads (Cat. LSKMAGAG02, Merck, Kenilworth, NJ, USA) for 1 hour at 4°C according to the manufacturer's instruction. After capturing magnetic beads, supernatants were collected followed by incubation of antibodies and samples with rotating overnight at 4°C. Magnetic beads were additionally added to the immune complexes, and then incubated at room temperature for 15 min with rotation. The immune complexes were washed three times in washing buffer, resuspended in the elution buffer and heated at 70 °C for 10 minutes. The complexes were evaluated by western blot analysis. 10% of total protein was used as an input control to determine the purity of each protein.

## Supplementary Figures

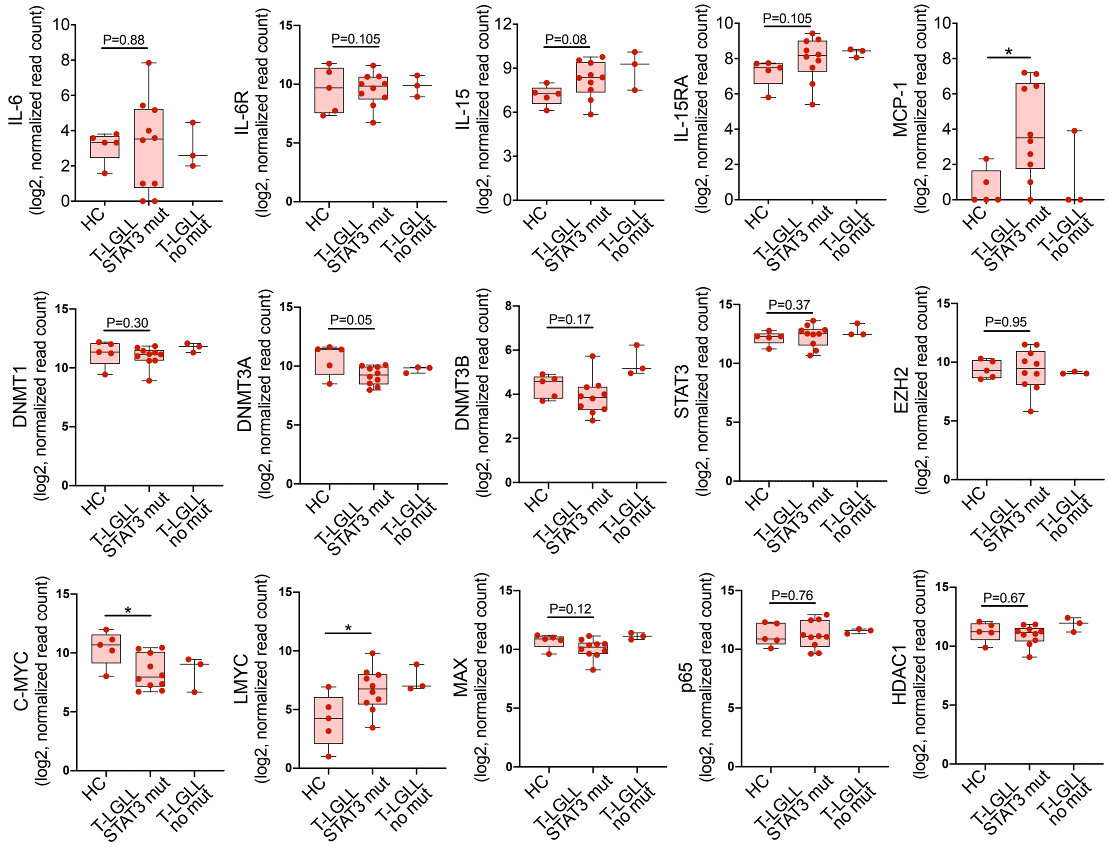


**Supplementary Figure 1.** RNA-seq data in CD8+ T cells of T-LGLL patients and healthy controls. RNA-seq data of IL-6, IL6R, IL-15, IL-15RA, MCP-1, DNMT1, DNMT3A, DNMT3B, STAT3, EZH2, MYC, MYCL, MAX, p65 and HDAC1 in CD8+ T cells of T-LGLL patients harboring STAT3 mutation (T-LGLL STAT3 mut, n = 10), patients without mutations (T-LGLL no mut, n = 3) and healthy controls (HC, n = 5). Each dot represents one individual. Data are expressed as mean ± SD, and statistically significant difference was evaluated using Mann-Whitney U test. *, P < 0.05.

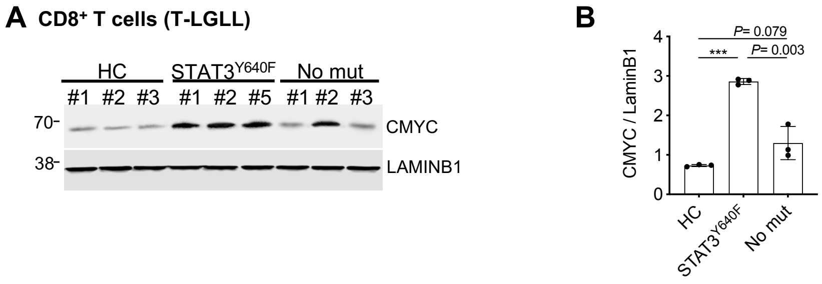


**Supplementary Figure 2**. **MYC protein expression of CD8+ T cells from healthy controls (HC) and T-LGLL patients harboring *STAT3$^{Y640F}$* mutation (STAT3$^{Y640F}$) and T-LGLL patients (No mut) without STAT3 mutations**. **(A)** Western blot analysis was performed with C-MYC specific antibody. LaminB1 served as a loading control. **(B)** Quantitative presentation of the western blot assays using ImageJ software (version 2.0.0). P values are derived from unpaired t-test using GraphPad Prism (Ver8.3.0). Error bar present Mean ± SD (n=3 per group). ***, P < 0.001.

A

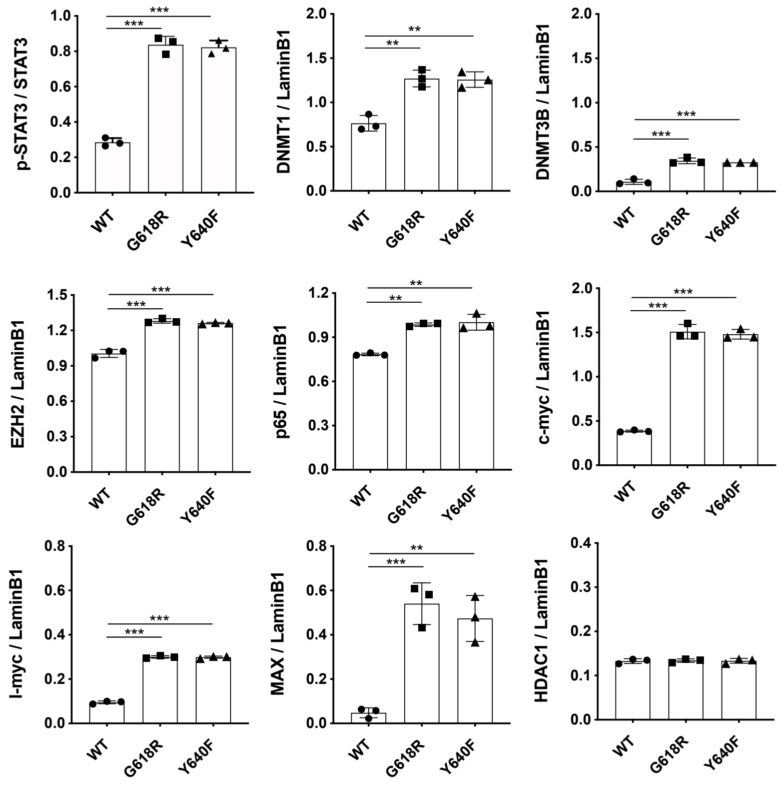


B

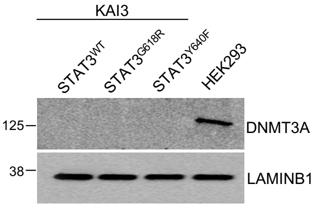


**Supplementary Figure 3. Protein expression with quantitative presentation in KAI3 NK cells. (A)** Quantitative presentation of the protein expression level presented in Figure 2C. Protein levels were normalized with LaminB1, a protein loading control. Quantitative presentation of the western blot assays using ImageJ software (version 2.0.0). P values are derived from unpaired t-test using GraphPad Prism (Ver8.3.0). Error bar present Mean ± SD (n=3 per group). *, $P < 0.05$; **, $P < 0.01$; ***, $P < 0.001$. **(B)** DNMT3A expression in KAI3 cells. HEK293 was used as a positive control. LAMINB1, a loading control.

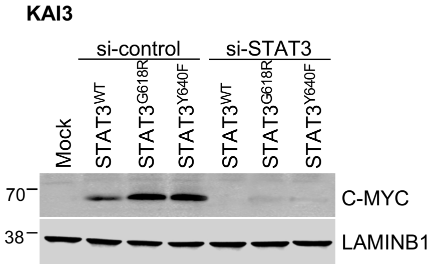


**Supplementary Figure 4. STAT3 knockdown decreases C-MYC expression in KAI3 NK cells.** KAI3 NK cells (STAT3WT, STAT3G618R and STAT3Y640F) were transfected with STAT3-siRNA (si-STAT3) and control siRNA (si-con) for 72 h followed by serum starvation for 12 hours. Western blot analysis was performed with pYSTAT3, STAT3 and C-MYC specific antibodies. LaminB1 served as loading control and data is representative of three independent experiments.

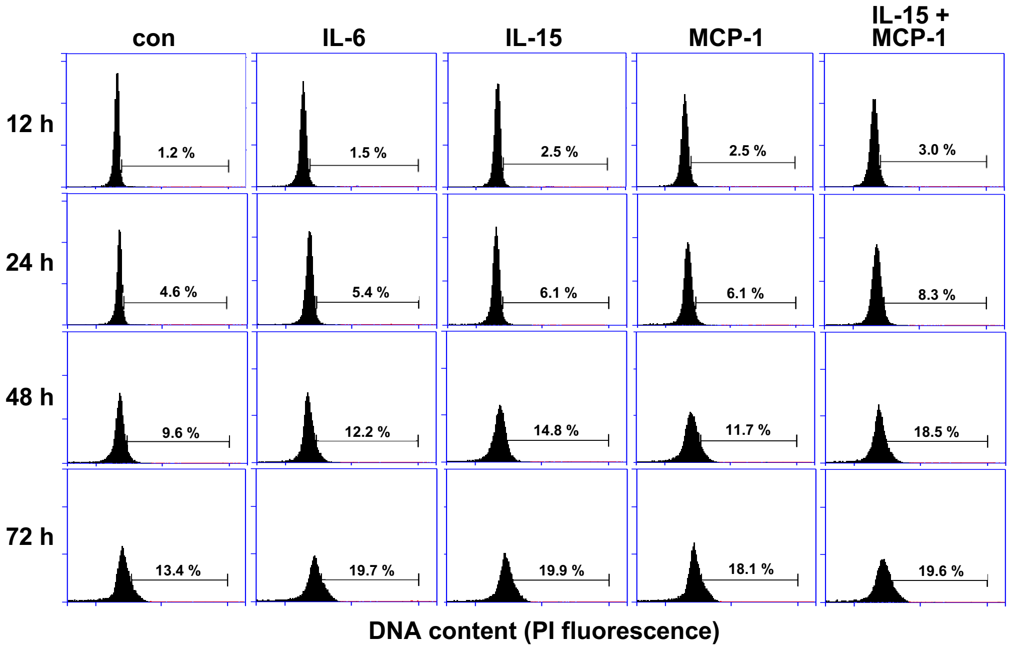


**Supplementary Figure 5. CD8+ T cell proliferation in response to IL-6, IL-15 and MCP-1.** CD8+ T cells from healthy controls were incubated with IL-6, IL-15 and/or MCP-1 for 12 hours. Cells were stained with propidium iodide (PI). Cell proliferation was identified through S/G2/M phases as percentages. The data was analyzed using BD Accuri™ C6 Plus. Data is representative of three independent experiments.

A

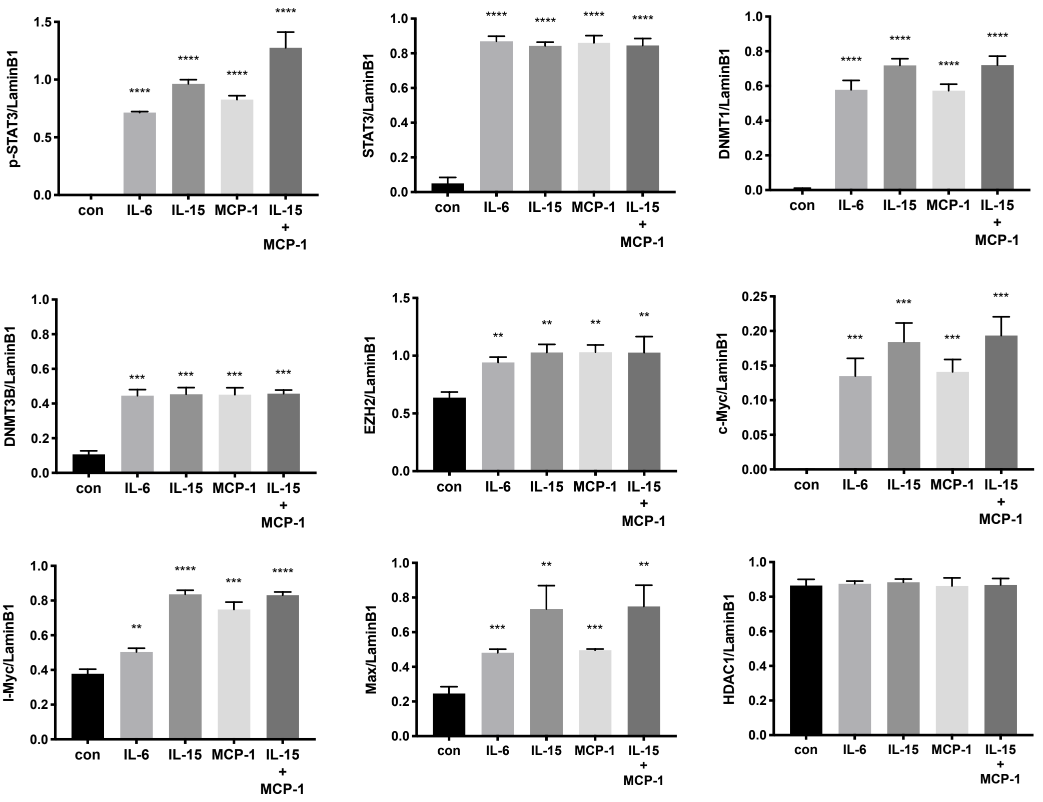


B

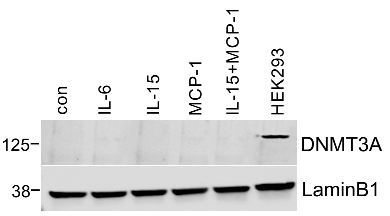


**Supplementary Figure 6**. **Protein expression with quantitative presentation in cytokine stimulated healthy CD8+ T cells. (A)** Quantitative presentation of the protein expression level in Figure 3A. Protein levels were normalized with LaminB1, a protein loading control. Quantitative presentation of the western blot assays using ImageJ software (version 2.0.0). P values are derived from unpaired t-test using GraphPad Prism (Ver8.3.0). Error bar present Mean ± SD (n=3 per group). *, $P < 0.05$; **, $P < 0.01$; ***, $P < 0.001$; ****, $P < 0.0001$. **(B)** DNMT3A expression in CD8+ T cells. HEK293 was used as a positive control. LAMINB1, a loading control.

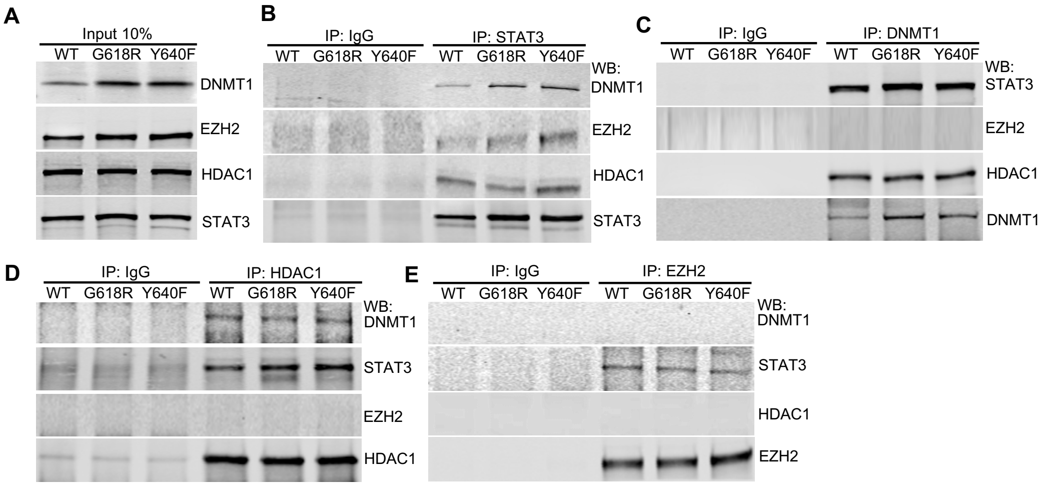


**Supplementary Figure 7**. **Physical interaction of STAT3 with DNMT1, EZH2, and HDAC1 in KAI3 NK cells.** Co-Immunoprecipitation (Co-IP) analysis of the nuclear protein lysates from KAI3 cells expressing *STAT3* wildtype, *STAT3* G618R and *STAT3* Y640F. KAI3 NK cells were serum starved for 12 h followed by nuclear extraction. **(A)** The nuclear extracts (10%) of the cells were used as input control. Co-IP was performed with antibody against **(B)** STAT3, **(C)** DNMT1, **(D)** HDAC1 and **(E)** EZH2. Rabbit IgG Isotype control (IgG), a negative control for non-specific binding for Co-IP. Immunoprecipitated complexes were probed using the antibodies as indicated.

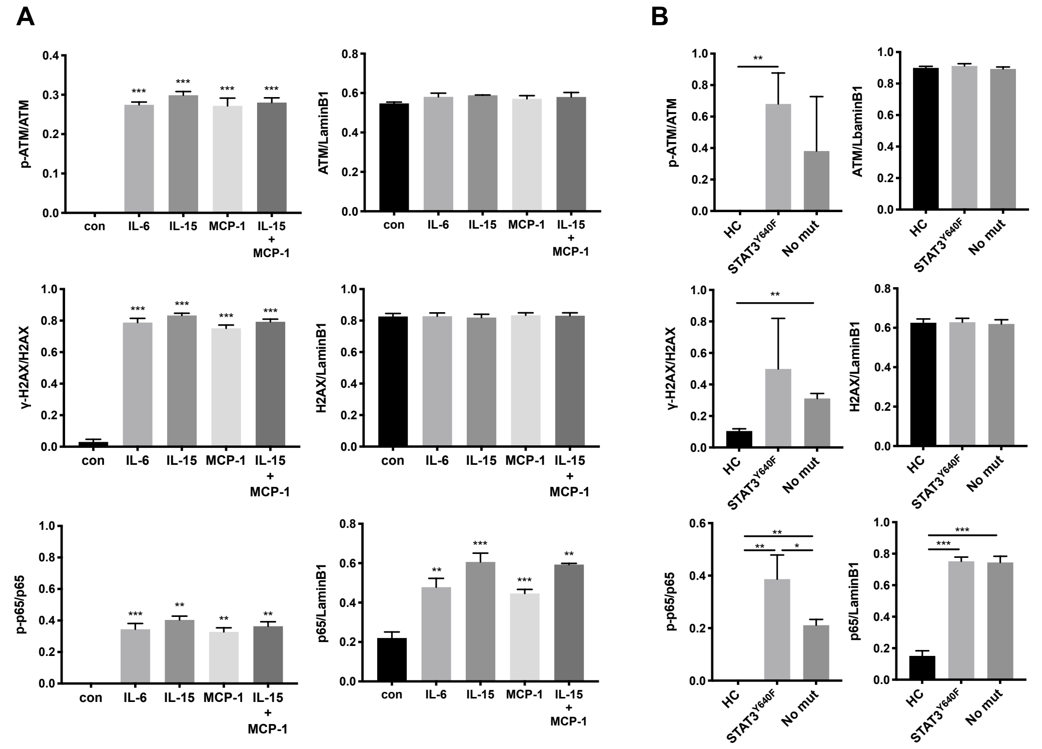


**Supplementary Figure 8. Quantitative presentation of the protein expression level presented in Figure 5D-E. (A)** Quantitative presentation of the protein expression level presented in Figure 5D. LaminB1, a protein loading control. Data are representative of three independent experiments data. Quantification was performed using ImageJ software (version 2.0.0). P values are derived from unpaired t-test using GraphPad Prism (Ver8.3.0). Error bar present Mean ± SD (n=3 per group). *, $P < 0.05$; **, $P < 0.01$; ***, $P < 0.001$. **(B)** Quantitative presentation of the protein expression level presented in Figure 5E.

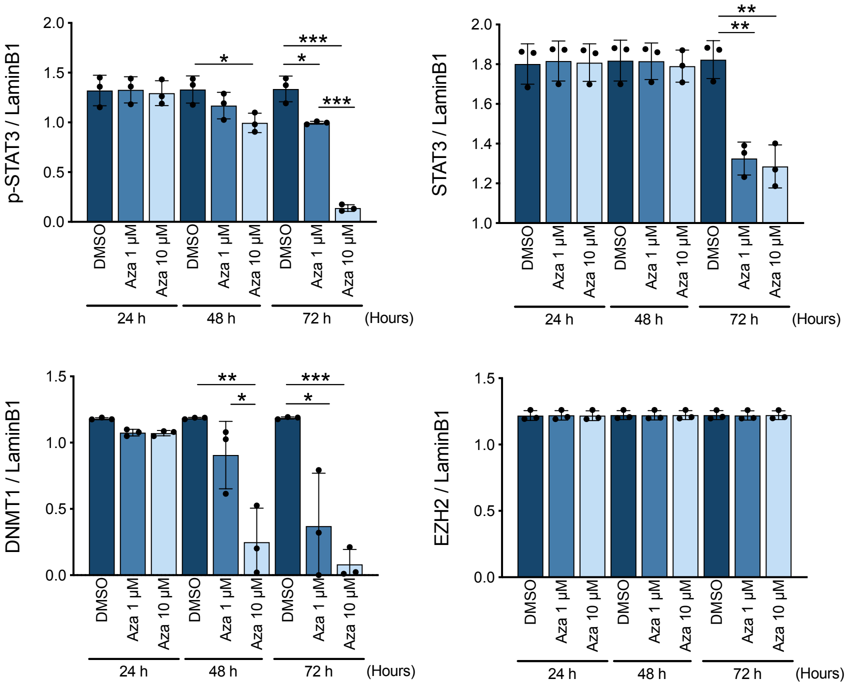


**Supplementary Figure 9. Quantitative presentation of the protein expression level presented in Figure 6B.** Protein levels were normalized with LaminB1, a protein loading control. Quantitative presentation of the western blot assays using ImageJ software (version 2.0.0). P values are derived from unpaired t-test using GraphPad Prism (Ver8.3.0). Error bar present Mean ± SD (n = 3). *, $P < 0.05$; **, $P < 0.01$; ***, $P < 0.001$.

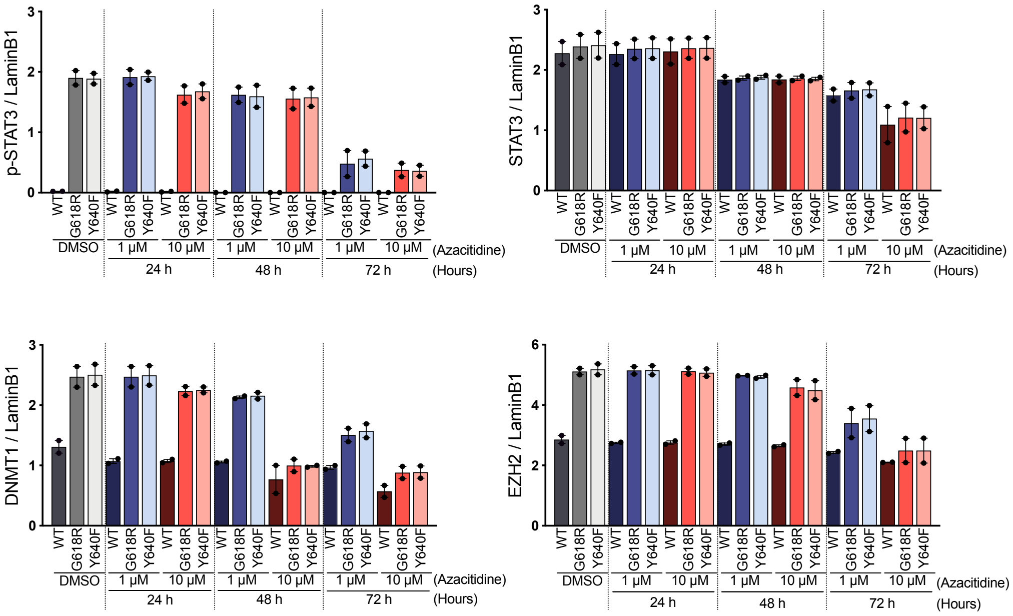


**Supplementary Figure 10 . Quantitative presentation of the protein expression level presented in Figure 6D.** KAI3 cells were treated with azacitidine for 24, 48 and 72 hours. Protein levels were normalized with LaminB1, a protein loading control. Each dot represents the quantified protein from one experiment. Error bar indicates range (n = 2). Quantitative presentation of the western blot assays using ImageJ software (version 2.0.0).

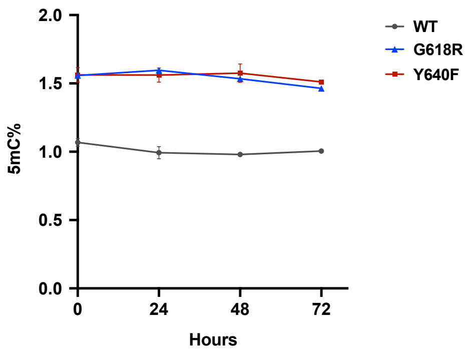


**Supplementary Figure 11. Global methylation level in KAI3 cells cultured with DMSO.** KAI3 cells expressing *STAT3* wildtype (WT, n=3), *STAT3*$^{G618R}$ (G618R, n=3) and *STAT3*$^{Y640F}$ (Y640F, n=3) were cultured in RPMI1640 including IL-2 (25IU/ml) at 24, 48, and 72 h. %5-mC were evaluated by fluorescence according to manufacturer's instruction. Error bar present mean ± SD (n=3 per group).

**A** KAI3

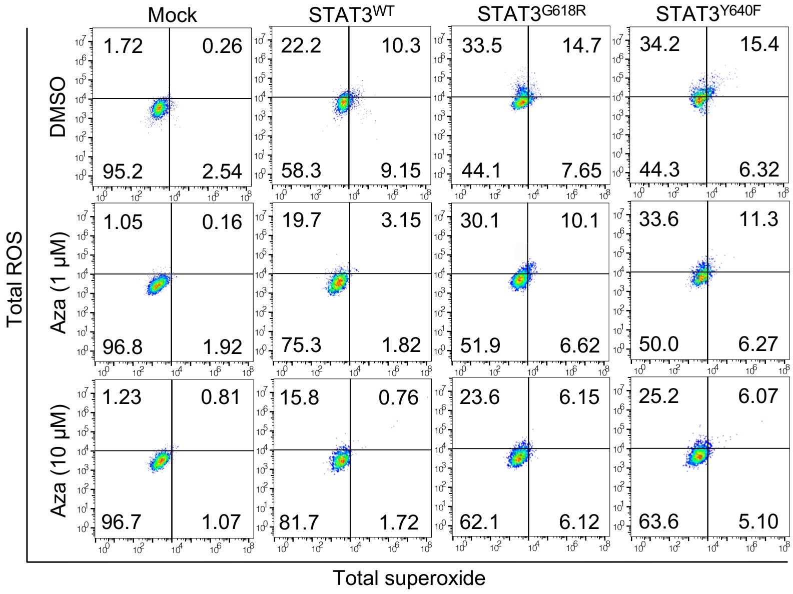
Mock
STAT3WT
STAT3G618R
STAT3Y640F
DMSO
Aza (1 μM)
Aza (10 μM)
Total ROS
Total superoxide
1.72
0.26
95.2
2.54
22.2
10.3
58.3
9.15
33.5
14.7
44.1
7.65
34.2
15.4
44.3
6.32
1.05
0.16
96.8
1.92
19.7
3.15
75.3
1.82
30.1
10.1
51.9
6.62
33.6
11.3
50.0
6.27
1.23
0.81
96.7
1.07
15.8
0.76
81.7
1.72
23.6
6.15
62.1
6.12
25.2
6.07
63.6
5.10

**B** KAI3

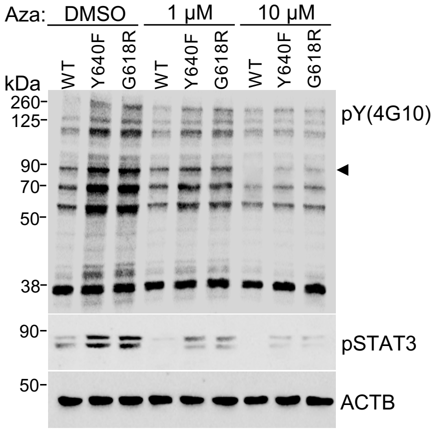
Aza:
DMSO
1 μM
10 μM
WT
Y640F
G618R
WT
Y640F
G618R
WT
Y640F
G618R
kDa
260-
125-
90-
70-
50-
38-
90-
50-
pY(4G10)
pSTAT3
ACTB

**Supplementary Figure 12. Azacitidine decreased ROS and tyrosine phosphorylation in KAI3 NK cells. (A)** Flow cytometry analysis to examine the expression of total ROS and superoxide in KAI3 cells stably expressing *STAT3*$^{WT}$, *STAT3*$^{G618R}$ and *STAT3*$^{Y640F}$ presented in Figure 6F. Cells were cultured in RPMI1640 including limited amout of IL-2 (25/IUml) in the presence of azacytidine (1 μM or 10 μM) or absence (DMSO alone) for 72 hours. Cells were labeled with ROS-fluorescent dye and superoxide-fluorescent dye, and then analyzed using flow cytometer. KAI3 cells expressing empty vector (Mock) were used as a control. Data are representative of three independent experiments data. **(B)** Western blot of whole cell tyrosine phosphorylation (4G10). KAI3 cells treated with azacitidine (1 μM or 10 μM) for 72 hours followed by Western blot analysis with anti-phosphotyrosine (4G10), anti-STAT3 and anti-ATCB. The major tyrosine-phosphorylated bands induced by STAT3 GOF variants (Y705) are indicated by arrow.

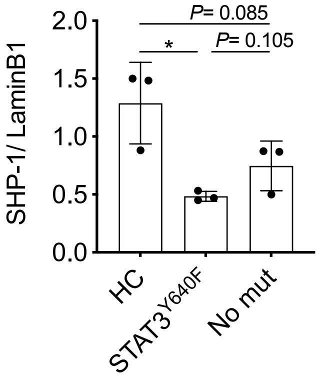


**Supplementary Figure 13. Quantitative presentation of the protein expression level presented in Figure 7A.** SHP1 protein level was normalized with LaminB1, a protein loading control. Quantitative presentation of the western blot assays using ImageJ software (version 2.0.0). P values are derived from unpaired t-test using GraphPad Prism (Ver8.3.0). Error bar present Mean ± SD (n = 3). *, P < 0.05.

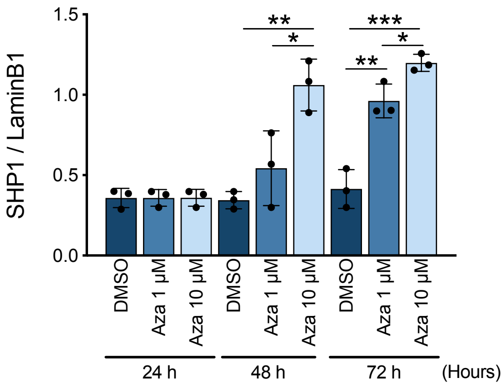


**Supplementary Figure 14. Quantitative presentation of the protein expression level presented in Figure 7B.** SHP1 protein level was normalized with LaminB1, a protein loading control. Quantitative presentation of the western blot assays using ImageJ software (version 2.0.0). P values are derived from unpaired t-test using GraphPad Prism (Ver8.3.0). Error bar present Mean ± SD (n = 3). *, $P < 0.05$; **, $P < 0.01$; ***, $P < 0.001$.

# Supplementary Tables

## Supplementary Table 1. Patients clinical characteristics

| Patient | Age at dg | Sex | Treatment | Vbeta | STAT3 mutation | Application in the study |
|---|---|---|---|---|---|---|
| T-LGLL (*STAT3*$^{Mut}$) 1 | 67 | F | Methorexae | CD8+ (Vb7.2: 63%, Vb23: 10%) | Y640F (VAF 25%) | CK, qPCR, WB, 5mC, ROS, DSRT |
| T-LGLL (*STAT3*$^{Mut}$) 2 | 53 | F | Prednison, Hydroxyclorokine | CD8+ (Vb8: 36%) | Y640F (VAF 16%) | CK, qPCR, WB, 5mC, DSRT |
| T-LGLL (*STAT3*$^{Mut}$) 3 | 70 | M | No | CD8+ (Vb1: 34%, Vb8: 16%) | Y640F (VAF 3.5%) | qPCR, WB, 5mC |
| T-LGLL (*STAT3*$^{Mut}$) 4 | 70 | M | Methorexate, Hydroxyclorokine | CD8+ (Vb13.1: 10%, Vb13.2: 17.6%, Vb14: 13.5%) | Y640F (VAF 6%) | qPCR, WB, 5mC, ROS |
| T-LGLL (*STAT3*$^{Mut}$) 5 | 69 | F | No | CD8+ (Vb7.1: 77.9%) | Y640F (VAF 36%) | qPCR, WB, 5mC, ROS, DSRT |
| T-LGLL (*STAT3*$^{Mut}$) 6 | 58 | F | No | CD8+ (Vb4: 14.8%, Vb21.3: 24%) | Y640F (VAF 3%) | CK |
| T-LGLL (*STAT3*$^{Mut}$) 7 | 57 | M | Oxiclorin, Methotrexate | CD8+ (Vb14: 5.1%) | Y657ins (VAF 5.9%) | CK |
| T-LGLL (*STAT3*$^{Mut}$) 8 | 59 | F | No | 90% CD8+ of CD3+. No clear expansions | D661Y (VAF 37%) | CK, qPCR |
| T-LGLL (*No STAT3*$^{Mut}$) 1 | 65 | M | Methylprednisolone | CD8+ (Vb8: 8.36%, Vb22: 8.52%) | No mutations | qPCR, WB, 5mC |
| T-LGLL (*No STAT3*$^{Mut}$) 2 | 75 | M | Prednisolone | CD8+ (Vb8: 18.4%) | No mutations | qPCR, WB, 5mC |
| T-LGLL (*No STAT3*$^{Mut}$) 3 | 66 | F | No | CD8+ (Vb14: 21%) | No mutations | CK, WB, 5mC |
| T-LGLL (*No STAT3*$^{Mut}$) 4 | 54 | M | Cyclosporine | CD8+ (Vb4: 8.4%, Vb13.6: 7.8%, Vb14: 7.1%) | No mutations | CK, qPCR, WB, 5mC |
| T-LGLL (*No STAT3*$^{Mut}$) 5 | 50 | F | Cyclosporine | CD8+ (Vb17: 17%) | No mutations | CK |
| T-LGLL (*No STAT3*$^{Mut}$) 6 | 40 | M | No | CD8+ (Vb13.2: 69%) | No mutations | qPCR, WB, 5mC, ROS |
| T-LGLL (*No STAT3*$^{Mut}$) 7 | 59 | M | No | CD8+ (Vb3: 89%) | No mutations | CK, qPCR, 5mC, ROS |
| T-LGLL (*No STAT3*$^{Mut}$) 8 | 61 | M | No | CD8+ (Vb8: 6.1%) | No mutations | qPCR, 5mC, ROS |
| T-LGLL (*No STAT3*$^{Mut}$) 9 | 56 | M | Methotrexate, Prednison | No clear expansions. | No mutations | 5mC |
| T-LGLL (*No STAT3*$^{Mut}$) 10 | 71 | M | Methotrexate | No clear expansions | No mutations | 5mC |

STAT3$^{Mut}$, Patients harboring *STAT3* mutations; No STAT3$^{Mut}$, Patients without *STAT3* mutations; M, Male; F, Female; VAF, Variant allele frequency; CK, Olink-Serum Cytokine measurement; WB, Western blot assay; 5mC, Methylation quantification; ROS, Reactive oxygen species measurement; DSRT, Drug sensitivity and resistant test.

**Supplementary Table 2. Sequence of siRNA**

| Target gene | Sequence |
|---|---|
| STAT3 | 5’-GGAGAAGCAUCGUGAGUGA-3’ |
| SHP1 | 5’-GGAACAAAUGCGUCCCAUA-3’ |
| Control | 5’-UAGCGACUAAACACAUCAA-3’ |

## Supplementary Table 3. Olink-Serum Cytokine measurement

| | LGLL (n = 9) (Median) | HC (n = 8) (Median) | $Log_2$Fold change | P-value (P<0.05) | FDR |
|---|---|---|---|---|---|
| MIP-1 alpha | 4.1 | 2.2 | 1.74583 | 0.000382 | 0.0154518 |
| TNFSF14 | 2.7 | 1.75 | 1.23889 | 0.000661 | 0.0154518 |
| CCL4 | 6.9 | 5 | 1.66528 | 0.000792 | 0.0154518 |
| CDCP1 | 3.3 | 1.9 | 1.60694 | 0.000846 | 0.0154518 |
| PD-L1 | 5.5 | 4.25 | 1.07917 | 0.000849 | 0.0154518 |
| MCP-3 | 1.8 | 0.7 | 1.06667 | 0.00163 | 0.024721667 |
| TNFRSF9 | 7.4 | 5.8 | 1.55972 | 0.002215 | 0.028795 |
| CXCL10 | 10.9 | 8.55 | 2.09444 | 0.003353 | 0.036268556 |
| IL-6 | 4.6 | 2.45 | 2.65139 | 0.003587 | 0.036268556 |
| IL-15RA | 1.2 | 0.35 | 0.854167 | 0.004602 | 0.039700818 |
| IL-18R1 | 7.5 | 6.85 | 0.779167 | 0.004799 | 0.039700818 |
| CXCL11 | 8.5 | 6.1 | 2.52639 | 0.007479 | 0.049725 |
| IL-18 | 10.2 | 8 | 1.59583 | 0.007488 | 0.049725 |
| CCL20 | 7.5 | 5.9 | 1.40972 | 0.00765 | 0.049725 |
| IL-8 | 6.9 | 5.2 | 1.4875 | 0.011178 | 0.0647465 |
| MCP-1 | 9.3 | 8.5 | 0.836111 | 0.011384 | 0.0647465 |
| IL-10 | 4.2 | 2.6 | 1.84028 | 0.014177 | 0.075888647 |
| LAP TGF-beta-1 | 7.3 | 6.35 | 0.743056 | 0.015901 | 0.080388389 |
| CD5 | 4.8 | 4.05 | 0.904167 | 0.017564 | 0.084122316 |
| OPG | 10.5 | 9.85 | 0.629167 | 0.020925 | 0.091173333 |
| VEGF-A | 10.7 | 9.95 | 0.705556 | 0.02104 | 0.091173333 |
| IL-10RB | 7.5 | 7.1 | 0.409722 | 0.022342 | 0.092414636 |
| CASP-8 | 2.9 | 1.65 | 1.05417 | 0.027303 | 0.1000965 |
| HGF | 7.5 | 7.15 | 0.756944 | 0.027545 | 0.1000965 |
| IL-2RB | 1.3 | 1.3 | 0.738889 | 0.028525 | 0.1000965 |
| IL-10RA | 1.6 | 1.15 | 0.902778 | 0.028599 | 0.1000965 |
| SCF | 8.8 | 9.35 | -0.776389 | 0.030769 | 0.101998 |
| uPA | 10.9 | 10.4 | 0.566667 | 0.031384 | 0.101998 |
| CCL11 | 8.1 | 7.5 | 0.568056 | 0.034484 | 0.104391219 |
| FGF-21 | 2.8 | 1.15 | 1.46667 | 0.036245 | 0.104391219 |
| ADA | 6.7 | 5.55 | 0.861111 | 0.036259 | 0.104391219 |
| CXCL9 | 8.9 | 6.95 | 1.82361 | 0.036709 | 0.104391219 |
| CD6 | 5.9 | 4.4 | 1.25417 | 0.039844 | 0.109872848 |
| CCL19 | 10 | 8.35 | 1.17083 | 0.047641 | 0.1240824 |
| SLAMF1 | 3.1 | 2.15 | 0.747222 | 0.047724 | 0.1240824 |

| | LGLL (n = 9) (Median) | HC (n = 8) (Median) | $Log_2$Fold change | P-value (P>0.05) | FDR |
|---|---|---|---|---|---|
| FGF-5 | 1.7 | 1.35 | 0.279167 | 0.053621 | 0.135541972 |
| NRTN | 0.9 | 0 | 1.90833 | 0.057343 | 0.138422974 |
| TRAIL | 8.7 | 8.4 | 0.338889 | 0.057803 | 0.138422974 |
| CD244 | 7.6 | 6.5 | 0.893056 | 0.069896 | 0.163090667 |
| 4E-BP1 | 8.2 | 7.25 | 1.08333 | 0.074046 | 0.16845465 |
| hGDNF | 2.4 | 2.1 | 0.691667 | 0.084345 | 0.187204756 |
| CX3CL1 | 6.8 | 6.3 | 0.895833 | 0.087708 | 0.190034 |
| OSM | 2.4 | 1.95 | 0.608333 | 0.093198 | 0.195951955 |
| IL-17A | 0 | 0 | 0.326389 | 0.094746 | 0.195951955 |
| SIRT2 | 4.2 | 3.3 | 1.62083 | 0.099318 | 0.200018 |
| IL-12B | 6.1 | 4.65 | 0.769444 | 0.102255 | 0.200018 |
| MMP-10 | 9 | 9.35 | -0.566667 | 0.103306 | 0.200018 |
| IL-20 | 0 | 0 | 0.977778 | 0.123121 | 0.233416896 |
| IL-17C | 1.2 | 0.55 | 0.518056 | 0.146246 | 0.271599714 |
| Beta-NGF | 1.2 | 1 | 0.209722 | 0.157826 | 0.284303627 |
| ARTN | 0 | 0 | 0.0888889 | 0.159335 | 0.284303627 |
| TGFA | 1.6 | 1.3 | 0.348611 | 0.1674 | 0.29295 |
| STAMPB | 3.7 | 3.45 | 1.06528 | 0.171199 | 0.293945453 |
| MCP-2 | 9.4 | 8.45 | 0.5625 | 0.184714 | 0.311277296 |
| MMP-1 | 2.3 | 1.25 | 0.8625 | 0.207696 | 0.343642473 |
| EN-RAGE | 2.6 | 1.95 | 1.05278 | 0.215438 | 0.34574085 |
| IL-24 | 0 | 0 | 1.45417 | 0.217127 | 0.34574085 |
| IFN-gamma | 0.4 | 0 | 0.940278 | 0.220449 | 0.34574085 |
| IL-13 | 0 | 0 | 0.563889 | 0.225876 | 0.34574085 |
| TNFB | 4.2 | 3.45 | 0.556944 | 0.227961 | 0.34574085 |
| FGF-19 | 7.3 | 8.55 | -0.7625 | 0.235658 | 0.351555377 |
| CD40 | 10.3 | 9.5 | 0.668056 | 0.256021 | 0.375772758 |
| NT-3 | 2.6 | 2.4 | 0.743056 | 0.27251 | 0.393625556 |
| CCL28 | 2.2 | 2.1 | 0.545833 | 0.291712 | 0.414778 |
| IL-1 alpha | 0 | 0 | 0.0625 | 0.303673 | 0.4251422 |
| IL-5 | 0 | 1.2 | -0.720833 | 0.321978 | 0.442147269 |
| DNER | 7.3 | 7.65 | -0.293056 | 0.325537 | 0.442147269 |
| LIF | 0 | 0 | 0.748611 | 0.333069 | 0.445724691 |
| AXIN1 | 2.7 | 2.3 | 0.790278 | 0.35049 | 0.462240435 |
| LIF-R | 7.4 | 7.15 | 0.168056 | 0.362152 | 0.464534493 |
| TSLP | 0 | 0 | 0.188889 | 0.362439 | 0.464534493 |
| TWEAK | 8.9 | 9.05 | -0.202778 | 0.370941 | 0.468828208 |
| TNF | 0 | 0 | 0.602778 | 0.389172 | 0.485132219 |
| IL-4 | 0.8 | 0.65 | 0.7125 | 0.396051 | 0.487035689 |
| FGF-23 | 3.3 | 2.75 | 0.458333 | 0.435794 | 0.528763387 |
| CXCL5 | 8.7 | 10.05 | -0.783333 | 0.448241 | 0.534409909 |
| Flt3L | 8.8 | 8.3 | 0.265278 | 0.452193 | 0.534409909 |
| MCP-4 | 2.6 | 2.55 | 0.245833 | 0.45909 | 0.535605 |
| IL-20RA | 0 | 0 | 0.0972222 | 0.488478 | 0.56267719 |
| ST1A1 | 1.6 | 0.85 | 0.543056 | 0.502538 | 0.571636975 |
| IL-33 | 0 | 0 | 0.701389 | 0.52898 | 0.594286173 |
| CXCL6 | 6.9 | 7 | 0.241667 | 0.566061 | 0.625007735 |
| TRANCE | 5.4 | 5.45 | 0.270833 | 0.570062 | 0.625007735 |
| CST5 | 6.3 | 6.15 | -0.180556 | 0.595962 | 0.6456255 |
| CXCL1 | 8 | 7.8 | 0.244444 | 0.6725 | 0.719970588 |
| CCL25 | 6.1 | 6.15 | -0.126389 | 0.689787 | 0.722491885 |
| IL-7 | 1.5 | 1.95 | -0.220833 | 0.690734 | 0.722491885 |
| CCL23 | 9.9 | 9.85 | 0.0791667 | 0.842717 | 0.871445989 |
| IL-2 | 0 | 0 | -0.0263889 | 0.882209 | 0.902033921 |
| BDNF | 4.1 | 6.15 | -0.284722 | 0.894061 | 0.903995011 |
| CSF-1 | 8.6 | 8.2 | -0.0402778 | 0.932164 | 0.932164 |

Plasma protein expression levels (NPX, $log_2$) presented as median. Significance was determined by Mann-Whitney U test. LGLL, Large granular lymphocytic leukemia patients; HC, Healthy controls; NPX, Normalized expression value; FDR, False discovery rate. The assay was performed at Olink Proteomics AB (www.olink.com, Uppsla, Sweden).

## Supplementary Table 4. Primer list of RT-qPCR

| Target gene | Sequence |
|---|---|
| *IL-2* forward | 5’-AACTCACCAGGATGCTCACATTTA-3’ |
| *IL-2* reverse | 5’-TCCCTGGGTCTTAAGTGAAAGTTT-3’ |
| *IL-4* forward | 5’-CGACTGCACAGCAGTTCCA-3’ |
| *IL-4* reverse | 5’-AGGTTCCTGTCGAGCCGTTT-3’ |
| *IL-6* forward | 5’-*GTAGCCGCCCCACACAGA*-3’ |
| *IL-6* reverse | 5’-*CATGTCTCCTTTCTCAGGGCTG*-3’ |
| *IL-15* forward | 5’-*CCATCCAGTGCTACTTGTGTTTACTT*-3’ |
| *IL-15* reverse | 5’-*CCAGTTGGCTTCTGTTTTAGGAA*-3’ |
| *MCP-1* forward | 5’-*ACTCTCGCCTCCAGCATGAA*-3’ |
| *MCP-1* reverse | 5’-TTGATTGCATCTGGCTGAGC-3’ |
| *β-actin* forward | 5’-*GTTGTCGACGACGAGCG*-3’ |
| *β-actin* reverse | 5’-*GCACAGAGCCTCGCCTTRT*-3’ |

## Supplementary Table 5. Drug sensitivity and resistence test data

| Sample | Cells | DRUG | ANALYSIS_ NAME | IC50 | SLOPE | MAX | MIN | Min.Conc. tested | Max.Conc. tested | IC50_std_error | D1 | D2 | D3 | D4 | D5 | DSS |
|---|---|---|---|---|---|---|---|---|---|---|---|---|---|---|---|---|
| Patient_1 | CD8+ T cells *STAT3* Y640F | Azacitidine | IC50 | 915 | 0,8 | 69,1 | 0 | 1 | 10000 | 1,4 | 15,9 | 4,3 | 15,9 | 28,1 | 69,1 | 7,7 |
| Patient_2 | CD8+ T cells *STAT3* Y640F | Azacitidine | IC50 | 305,9 | 0,8 | 73 | 0 | 1 | 10000 | 0,8 | -2,8 | -10 | 29,4 | 47,1 | 73 | 12,3 |
| Patient_3 | CD8+ T cells *STAT3* Y640F | Azacitidine | IC50 | 432,3 | 2,5 | 42,3 | 0 | 1 | 10000 | 2,2 | -13,7 | -8,5 | -17,2 | 42,3 | 33,5 | 6,8 |
| Healthy control_1 | CD8+ T cells *STAT3* Y640F | Azacitidine | IC50 | 10000 | 0,4 | 23,2 | 0 | 1 | 10000 | 148,3 | 1,4 | 15,9 | -20,4 | 23,2 | 6,6 | 0 |
| Healthy control_2 | CD8+ T cells *STAT3* Y640F | Azacitidine | IC50 | 10000 | 2,5 | 9,3 | 0 | 1 | 10000 | 5168,8 | 9,3 | -13,5 | 4,8 | -6,4 | 9,3 | 0 |
| Healthy control_3 | CD8+ T cells *STAT3* Y640F | Azacitidine | IC50 | 1888,7 | 2,5 | 29,5 | 0 | 1 | 10000 | 52 | -9,3 | -9,3 | 8,7 | 4,9 | 29,5 | 2,1 |
| KAI3_ *STAT3*_WT_1 | KAI3_ *STAT3*_WT | Azacitidine | IC50 | 1321,8 | 2,2 | 71,2 | 0 | 1 | 10000 | 1,1 | -7,3 | -2,3 | 3 | 24,9 | 71,2 | 7,2 |
| KAI3_ *STAT3*_WT_2 | KAI3_ *STAT3*_WT | Azacitidine | IC50 | 1369,1 | 2,5 | 74,4 | 0 | 1 | 10000 | 5,3 | -3,6 | -3,5 | -17,5 | 23,4 | 74,4 | 7,5 |
| KAI3_ *STAT3*_Y640F_1 | KAI3_ *STAT3*_Y640F | Azacitidine | IC50 | 1149 | 1,9 | 88,3 | 0 | 1 | 10000 | 0,3 | -1,5 | -10,6 | 3,6 | 38,2 | 88,3 | 9,5 |
| KAI3_ *STAT3*_Y640F_2 | KAI3_ *STAT3*_Y640F | Azacitidine | IC50 | 1191,7 | 1,3 | 88,3 | 0 | 1 | 10000 | 0,5 | 12,6 | 5,7 | 10,7 | 36,8 | 88,3 | 9,1 |
| KAI3_ *STAT3*_G618R_1 | KAI3_ *STAT3*_G618R | Azacitidine | IC50 | 1264,8 | 2,5 | 87,7 | 0 | 1 | 10000 | 2,3 | -3 | -10,2 | -5,6 | 31,4 | 87,7 | 9,2 |
| KAI3_ *STAT3*_G618R_2 | KAI3_ *STAT3*_G618R | Azacitidine | IC50 | 1213,4 | 2,5 | 90,5 | 0 | 1 | 10000 | 1 | -3,5 | -8,7 | 0,9 | 34,5 | 90,5 | 9,7 |

Drug: Azacitidine
Mechanism/Targets: Nucleoside analog DNA methyl transferase inhibitor
Class.explained: E. Differentiating/ epigenetic modifier
High.phase/Approval.status: Approved
Alias: 5-azacytidine,5-AzaC
activity.modifier: Prodrug, activated in the cells
Solvent: DMSO
High.conc.(nM): 10,000

**References**

1. Kim D, Park G, Huuhtanen J, Lundgren S, Khajuria RK, Hurtado AM, et al. Somatic mTOR mutation in clonally expanded T lymphocytes associated with chronic graft versus host disease. Nat Commun. 2020;11(1):2246.

2. Koskela HL, Eldfors S, Ellonen P, van Adrichem AJ, Kuusanmaki H, Andersson EI, et al. Somatic STAT3 mutations in large granular lymphocytic leukemia. N Engl J Med. 2012;366(20):1905-13.
